# Arabinogalactan Protein-Like Proteins From *Ulva lactuca* Activate Immune Responses and Plant Resistance in an Oilseed Crop

**DOI:** 10.3389/fpls.2022.893858

**Published:** 2022-05-20

**Authors:** Tereza Přerovská, Barbora Jindřichová, Svatopluk Henke, Jean-Claude Yvin, Vincent Ferrieres, Lenka Burketová, Petra Lipovová, Eric Nguema-Ona

**Affiliations:** ^1^Ecole Nationale Supérieure de Chimie de Rennes, CNRS, ISCR-UMR 6226, Univ Rennes, Rennes, France; ^2^Laboratory of Pathological Plant Physiology, Institute of Experimental Botany of the Czech Academy of Sciences, Prague, Czechia; ^3^Department of Biochemistry and Microbiology, University of Chemistry and Technology Prague, Prague, Czechia; ^4^Agro Innovation International TIMAC AGRO, Laboratoire de Nutrition Végétale, Pôle Stress Biotique, Saint Malo, France

**Keywords:** Arabinogalactan proteins, plant defense, elicitor, hemibiotrophic fungus, plant immunity, *Ulva lactuca*

## Abstract

Natural compounds isolated from macroalgae are promising, ecofriendly, and multifunctional bioinoculants, which have been tested and used in agriculture. Ulvans, for instance, one of the major polysaccharides present in *Ulva* spp. cell walls, have been tested for their plant growth-promoting properties as well as their ability to activate plant immune defense, on a large variety of crops. Recently, we have characterized for the first time an arabinogalactan protein-like (AGP-like) from *Ulva lactuca*, which exhibits several features associated to land plant AGPs. In land plant, AGPs were shown to play a role in several plant biological functions, including cell morphogenesis, reproduction, and plant-microbe interactions. Thus, isolated AGP-like proteins may be good candidates for either the plant growth-promoting properties or the activation of plant immune defense. Here, we have isolated an AGP-like enriched fraction from *Ulva lactuca* and we have evaluated its ability to (i) protect oilseed rape (*Brassica napus*) cotyledons against *Leptosphaeria maculans*, and (ii) its ability to activate immune responses. Preventive application of the *Ulva* AGP-like enriched fraction on oilseed rape, followed by cotyledon inoculation with the fungal hemibiotroph *L. maculans*, resulted in a major reduction of infection propagation. The noticed reduction correlated with an accumulation of H_2_O_2_ in treated cotyledons and with the activation of SA and ET signaling pathways in oilseed rape cotyledons. In parallel, an ulvan was also isolated from *Ulva lactuca*. Preventive application of ulvan also enhanced plant resistance against *L. maculans*. Surprisingly, reduction of infection severity was only observed at high concentration of ulvan. Here, no such significant changes in gene expression and H_2_O_2_ production were observed. Together, this study indicates that *U. lactuca* AGP-like glycoproteins exhibit promising elicitor activity and that plant eliciting properties of *Ulva* extract, might result not only from an ulvan-originated eliciting activities, but also AGP-like originated.

## Introduction

The whole agricultural sector is facing the forthcoming challenges to keep up productivity with a growing global population (Ray et al., 2013). Nowadays, high and continuous agricultural productivity is dependent on the use of chemical fertilizers and pesticides. Nevertheless, the excessive use of these compounds has adverse effects on human health and the environment (Carvalho, 2006). The recent progress in the use of either natural plant growth-promoting substances or microorganisms (also termed plant biostimulants) has allowed a reduction and optimized use of fertilizers. This combination of mineral nutrients and biostimulants allows a better nutrient use efficiency, a better crop tolerance against abiotic stresses, and indirectly, a better quality and an improved yield of the crops (Rouphael and Colla, 2020; du Jardin et al., 2020). Likewise, microorganisms/organisms or natural substances were also tested and used in agriculture as agents able to interfere by different means with the occurrence of plant diseases caused by pathogens. Natural substances encompass various types of biomolecules, which can be extracted from a vast number of plant species. Among these natural substances, plant elicitors are described as substances able to activate plant immune system, and further, to protect crop against various kind of pathogens and parasites both in conventional and organic agriculture (Wiesel et al., 2014; Jamiolkowska, 2020).

Plants have indeed developed an efficient immune system described in the zig-zag model from Jones and Dangl (2006), which can be triggered *via* (i) the perception of plant elicitors or pathogen/microbial/damage-associated molecular patterns (P/M/DAMPs) also known as pathogen-associated molecular pattern PAMP-triggered immunity (PTI) or (ii) specific pathogens’ effectors (effector-triggered immunity, ETI; Jones and Dangl, 2006). In field conditions, the mobilization of PTI by plant defence elicitors could lead to a pesticide reduction. In PTI, the recognition of plant elicitors by cell surface pattern-recognition receptors (PRRs) induces a series of early events, such as reactive oxygen species (ROS), nitric oxide production, and intracellular calcium influx. Then, intermediate events consist of activation of mitogen-activated protein kinases (MAPK) and phytohormone signaling (salicylic acid—SA, jasmonic acid—JA, and/or ethylene—ET; Bigeard et al., 2015). These signaling cascades trigger the induction of defense genes leading to the production of various defense-related compounds such as pathogensis-related (PR) proteins (Van Loon et al., 2006) or specialized antimicrobial compounds (Boller and Felix, 2009).

Many plant elicitors, also called PAMPs, which have been so far isolated and tested in both laboratory and field experiments, originated from microbes (MAMPs; e.g., flagellin; Zipfel et al., 2004), or plant themselves (DAMPs; oligogalacturonides; Hahn et al., 1981; Benedetti et al., 2015). A third category, called exogenous elicitors, which includes seaweed-based natural substances, was also reported to activate PTI. Interestingly, many macroalgae-based extracts were also reported to exhibit plant growth-promoting properties. Carrageenans are galactan-based polysaccharides commonly found in red macroalgae and were reported to exhibit plant-eliciting properties (Mercier et al., 2001). Laminarins, β-glucan-containing polysaccharides of brown macroalgae were also reported to exhibit plant-eliciting properties (Klarzynski et al., 2000; Aziz et al., 2003). Finally, ulvan polysaccharide, constitutive component of the cell walls of the green macroalgae *Ulva* genus was also reported to activate PTI (Cluzet et al., 2004; Jaulneau et al., 2010; Martin et al., 2020; Borba et al., 2021).

*Ulva* spp. belong to the class of Ulvophyceae, a group of green marine benthic algae, which dominates shallow marine environments and displays outstanding diversity regarding cytological and morphological characteristics (Wichard et al., 2015). *Ulva* spp. were shown to contain macro- and micronutrients, phytohormones, osmoprotectants, and other compounds with possible biological activities (Chbani et al., 2015; Shoubaky and Salem, 2016; Nabti et al., 2017). The presence of these compounds may accounts for well documented, *Ulva* extract-dependent, plant-growth promoting properties (Gireesh et al., 2011; Divya et al., 2015; Castellanos-Barriga et al., 2017; Paulert et al., 2021; Shefer et al., 2022). In addition to ulvans, *Ulva* spp. was also reported to contain fibrillar cellulose, mannan, or xylan polysaccharides in their cell walls (Domozych et al., 2012).

One class of proteins, the arabinogalactan proteins (AGPs), found in algal, moss, fern, and flowering plant cells walls and are strongly implicated in developmental processes (Lee et al., 2005; Nguema-Ona et al., 2012; Bartels and Classen, 2017; Renzaglia et al., 2017; Happ and Classen, 2019; Palacio-López et al., 2019) as well as in interaction with microorganisms (Nguema-Ona et al., 2013; Mareri et al., 2019). AGPs are proteoglycans consisting of two distinct moieties, the carbohydrate and the protein domain. The carbohydrate component typically accounts for 90%–98% of an AGP by weight and is rich in arabinose and galactose residues. The protein moiety, accounting for less than 10% of an AGP by weight is hydroxyproline-rich (Showalter, 2001; Seifert and Roberts, 2007). However, there is a wide range of variability in the structure and composition of both the carbohydrate and the polypetide parts. Based on the amino acid sequence and composition, AGPs were initially categorized into classical AGPs [consisting of a P/Hyp-rich domain heavily *O*-glycosylated, a hydrophobic C-terminal (C-ter) domain required for anchorage to the plasma membrane, and a signal peptide sequence] and non-classical AGPs (sometimes *N*-glycosylated and lacking the C-ter domain; Nguema-Ona et al., 2012). Non-classical AGPs also tend to be less heavily glycosylated (Showalter, 2001; Ma et al., 2018).

Although AGPs and AGP-like structures were reported to occur across the green and brown algae lineages, contrasting with the wealth of information available on land plant AGPs, much less is known about AGP occurrence, structure, and function in algae (Sørensen et al., 2011; Hervé et al., 2015; Palacio-López et al., 2019). Using immunocytochemistry and Yariv reagent, the presence of AGPs was described in several green microalgae of the freshwater-originated Charophyta division, specifically in Desmidiaceae, Coleochaetacea, Mesotaeniacea, Zygnemataceae, Chlorokybaceae, and Peniaceae families (Domozych et al., 2007, 2009; Eder et al., 2008; Sørensen et al., 2011; Palacio-López et al., 2019; Pfeifer et al., 2021). Furthermore, AGPs were detected as well in the Charale order, representing the multicellular algae with stem-like and leaf-like structures (Domozych et al., 2010). Within the Chlorophyta division, AGPs were also reported in Oedogoniaceae and Codiaceae families (Estevez et al., 2008, 2009; Fernández et al., 2010, 2015). Very recently, AGP-like glycoproteins were isolated for the first time from *Ulva lactuca* (Přerovská et al., 2021). In this study, *Ulva* AGP-like glycoproteins exhibited a contrasting reactivity with primary anti-AGP antibodies as well as with Yariv reagent when compared to AGP glycoproteins isolated from *Solanum lycopersicum*. While the amino acid analysis of the AGP-like glycoproteins purified by the β-d-glucosyl Yariv reagent showed a similarity between *Ulva* AGP-like glycoproteins and land plant AGPs, saccharide analysis revealed unique glycosylation of the *Ulva lactuca* AGP-like glycoproteins. Surprisingly, arabinose and galactose were not the most prevalent monosaccharides and the most outstanding was the presence of 3-*O*-methyl-hexose, which has never been described in the AGPs (Přerovská et al., 2021). Nevertheless, methylation of AGP glycans was previously reported (Bartels et al., 2017; Bartels and Classen, 2017; Happ and Classen, 2019; Temple et al., 2019; Pfeifer et al., 2020). Moreover, methylated glycoproteins and polysaccharides are widely distributed within algal cell walls (Ogawa et al., 2001; Bollig et al., 2007; Capek et al., 2008; Levy-Ontman et al., 2011; Staudacher, 2012; Mathieu-Rivet et al., 2014; Mócsai et al., 2019; Pfeifer and Classen 2020).

In the present study, an AGP-like enriched fraction from *Ulva lactuca* has been purified and chemically characterized. In order to find out if the *Ulva* AGP-like enriched fraction would exhibit plant eliciting properties, the fraction was tested for its ability to elicit the activation of PTI on oilseed rape (*Brassica napus*). Oilseed rape is widely grown in Europe, Canada, China, and Australia, and ranks second as oilseed production right after soybean (Neik et al., 2020; Raboanatahiry et al., 2021). Oilseed rape is confronted by a plethora of pathogenic agents, including *Plasmodiophora brassicae*, *Leptosphaeria maculans*, *Sclerotinia sclerotiorumm*, *Hyaloperonospora parasitica*, and others (Becker et al., 2017; Neik et al., 2017, 2020). *Leptosphaeria maculans* is a hemibiotrophic fungal pathogen causing blackleg disease, also called phoma stem canker (Lipková et al., 2021). The disease causes annually 10%–20% of yield losses (Van de Wouw and Howlett, 2020). The AGP-like enriched fraction was further tested for its ability to reduce the occurrence and the spread of blackleg disease on oilseed rape cotyledons. All along this work, the level of activation of PTI as well as the efficacy of the AGP-like enriched fraction was evaluated. In parallel, an ulvan was also isolated and its ability to both activate immune responses and to protect oilseed rape, was evaluated and compared to the AGP-like enriched fraction. Our results showed that the AGP-like enriched fraction was able to significantly activate PTI, and further, to protect the oilseed rape cotyledons from the occurrence and the spread of *L. maculans*. Interestingly, plants treated with the AGP-like enriched fraction showed a concentration-dependent reduction in the severity of *L. maculans* infection, while ulvan was effective only at the highest tested concentration. Likewise, the level of activation of PTI was more pronounced following the application of the AGP-like enriched fraction compared to the ulvan.

## Materials and Methods

### Biological Materials

*U. lactuca* materials collected in Brittany (France) were purchased from the European Marine Biological Resource Center (EMBRC, Station Biologique de Roscoff; https://embrc-france.obs-banyuls.fr) in 2017. *U. lactuca* was identified based on the sequence and phylogenetic analysis of rubisco large subunit (*rbcL*), internal transcribe spacer (ITS), and *tuf*A (plastid elongation factor) genes according to Vieira et al. (2016) and Lin et al. (2012). The material used for further extractions and analyses was freeze-dried and ground to a fine powder in CryoMill.

*B. napus* cultivar Columbus plants were grown hydroponically in perlite nourished with Steiner’s nutrient solution (Steiner, 1984) under controlled conditions (14/10 h, 22/20°C, day/night). For inoculation tests, gene expression tests and hydrogen peroxide detection cotyledon leaves were used.

The fungus *L. maculans* (anamorph *Phoma lingam*) isolate JN2 (Balesdent et al., 2001) was cultivated on V8 solidified medium (20% V8 vegetable juice, Campbell, 3 g·L^−1^ CaCO_3_, and 15 g·L^−1^ agar, autoclaved). Sporulation cultures and conidia suspension were prepared according to Šašek et al. (2012a). After harvesting, the spores were diluted to 10^8^ spore·ml^−1^ and stored at −20°C for a maximum of 6 months.

### Ulvan Extraction

Based on Yaich et al. (2013), 12.5 g of ground lyophilized *U. lactuca* was resuspended in 200 ml 50 mM HCl pH 2 and was incubated at 90°C for 3 h. After the extraction, the suspension was centrifuged for 10 min at 7,000 *g* at room temperature. The pH of the supernatant was adjusted to 3.5 by 1 M NaOH and precipitated overnight by three volumes of ethanol at 4°C. The pellet was obtained by centrifugation for 10 min at 7,000 *g* at 10°C, and the precipitate was washed three times by 50, 75, and 100% ethanol, centrifuged, dried, and lyophilized.

### Preparation of AGP-Like Enriched Fraction

1 g of ground freeze-dried *U. lactuca* was extracted with 4 ml of extraction buffer: 50 mM 4-morpholineethanesulfonic acid (MES) buffer pH 6, 0.2 M CaCl_2_, and 1 mM phenylmethylsulfonyl fluoride (PMSF). The extractions mixture was incubated 24 h at 4°C using a rotary mixer. Extraction mixture was centrifuged at 22,000 *g* for 20 min at 4°C.

For purification, the column XK 16/40 (GE Healthcare, United States) was fully packed by Q Sepharose® Fast Flow resin (GE Healthcare, United States). Subsequently, 50 ml of crude extract was 10 times diluted by 25 mM MES buffer pH 6 and left overnight at 4°C to precipitate. The AGP-like glycoproteins remained in the supernatant after the extract precipitation. The extract was then centrifuged, filtered by 0.45 μm, and loaded to the column by sample pump. The sample loading was followed by 200 ml 25 mM MES buffer pH 6 column wash, followed by 100 ml buffer with 0.2 M NaCl, and then step change to buffer with 0.5 M NaCl and after that linear gradient to 1.2 M NaCl on 150 ml. The next step was linear gradient 1.2–2 M NaCl in buffer on 50 ml. The column was reequilibrated by 200 ml wash with 2 M NaCl and 200 ml 25 mM MES buffer pH 6. The flow rate was 2.5 ml∙min^−1^. Localization of AGP-like glycoprotein in collected fractions was done by western blot assay and control for the presence of ulvan was done by TBO assay. Positive fractions were pulled together, desalted by dialysis using 100 kDa MWCO dialysis tubing (Repligen, United States) for 3 days against distilled water and lyophilized.

### Sodium Dodecylsulfate-Polyacrylamide Gel Electrophoresis and Western Blot

Samples were mixed with Laemmli sample buffer with a reducing agent, boiled for 10 min and 4–25 μl were loaded on 4%–15% Mini-PROTEAN® TGX Stain-Free™ precast polyacrylamide gels (Bio-Rad, United States). Gels were run at a constant current 200 V for approximately 35 min, and then they were stained by Pierce Silver Stain Kit (Thermo Fisher Scientific, United States). Separated proteins were transferred to the nitrocellulose membrane *via* the Trans-Blot Turbo system (Bio-Rad, United States), using the 10-min program for high molecular weight proteins, and were checked for the efficiency of transfer. The membrane was blocked with 5% low-fat milk in Tris-buffered saline (TBS) with 0.05% Tween 20 (v/v; TBST) overnight at 4°C on a rocking platform. JIM16 primary antibody (PlantProbes, United Kingdom) was used in 1:500 dilution in 5% low-fat milk in TBST for 1.5 h at room temperature on a rocking platform 100 rpm. After washing with TBST three times for 20 min at room temperature on a rocking platform, blots were incubated with an anti-rat IgG secondary antibody (Sigma Aldrich, United States) coupled to horseradish peroxidase in dilution 1:10,000 in 5% low-fat milk in TBST for 1.5 h at room temperature on the rocking platform 100 rpm. After washing as described before, the membranes were developed in SuperSignal West Femto Maximum Sensitivity Substrate (Thermo Fisher Scientific, United States) for 5 min at room temperature and the chemiluminescence was detected by ChemiDoc Imaging System (Bio-Rad, United States).

### FT-IR Analysis

FT-IR spectra (4,000–400 cm^−1^) were measured on Nicolet 6700 FT-IR spectrometer (Thermo Fisher Scientific, United States) using KBr tablets (transmission), 64 scans were accumulated with a spectral resolution of 2.0 cm^−1^. The spectra were smoothed, baseline-corrected and the normalization has been done in Omnic 8.0 (Thermo Fisher Scientific, United States). Finally, the spectra were exported in ASCII format to Origin Pro software (Microcal Origin, United States) for the preparation of graphs.

### Determination of Sulfated Polysaccharides by Toluidine Blue O

Based on Hahn et al. (2016), toluidine blue O (TBO) was dissolved in 20 mM maleic acid buffer pH 1 to a final concentration of 0.06 mmol·L^−1^. For measurement of calibration curves, ulvan (prepared according to the section “Ulvan Extraction”) and dextran sulfate in concentrations 0, 0.1, 0.25, 0.5, 0.75, and 1 mg·ml^−1^ were used. About 100 μl of calibration or sample solutions were mixed with 900 μl of TBO reagent, and the absorbance was measured at 632 nm. For the blank measurement was used distilled water.

### Determination of Protein Content by Bicinchoninic Acid Assay

Protein content was measured by Bicinchoninic Acid (BCA) Protein Macro Assay Kit (Serva, DE) according to the product manual. Briefly, for measurement of calibration line, the bovine serum albumin in concentrations 0, 0.025, 0.05, 0.1, 0.25, 0.5, 0.75, and 1 mg·ml^−1^ was used. About 50 μl of standards or samples were mixed with 1 ml of BCA reagent. In blank measurement was used just distilled water. The solutions were incubated at 37°C for 30 min and absorbance was read at 562 nm.

### Determination of Total Saccharide Content by Anthrone Assay

Based on Yemm and Willis (1954), anthrone reagent was prepared by dissolving 0.2 g of anthrone in a mixture of 5 ml of ethanol and 95 ml of 75% sulfuric acid on ice. For measurement of calibration line, glucose in concentrations 0, 0.01, 0.1, 1, 10, and 100 μg∙ml^−1^ was used. The 100 μl of samples (0.1 mg∙ml^−1^) or calibration solutions were mixed with 500 μl of anthrone reagent on ice. Afterward, the mixture was incubated for 10 min at 100°C, chilled on ice, and the saccharide content was determined spectrophotometrically at 625 nm.

### Determination of Uronic Acid Content

Based on Blumenkrantz and Asboe-Hansen (1973), galacturonic acid was used for measurement of the calibration line in concentrations 0, 40, 80, 120, 160, 200, and 240 μg∙ml^−1^. Samples (1 mg∙ml^−1^) and calibration solutions were diluted by distilled water 1:4 to final volume 500 μl and 3 ml of 12.5 mM sodium tetraborate decahydrate (0.478 g dissolved in 100 ml of 96% sulfuric acid) was added and the mixture was vortexed. The tubes were kept at 100°C for 5 min, chilled on ice and 50 μl of 0.15% (w/v) 3-hydroxybiphenyl in 0.5% NaOH was added. In the case of individual sample blank measurements, the use of 3-hydroxybiphenyl was omitted and only 0.5% NaOH was added. The solutions were vortexed and kept at room temperature for 30 min. The absorbance was measured at 520 nm. From the samples control solution of β-glucan (1 mg∙mL^−1^), as correction of neutral saccharide interference, was also subtracted.

### Saccharide Composition Analysis by High-Performance Anion-Exchange Chromatography

1 mg of samples were dissolved in 1 ml of 1 M H_2_SO_4_ and were hydrolyzed for 8 h at 90°C. To neutralize the samples, 300 mg of BaCO_3_ were added and incubated overnight on vortex. Samples were centrifuged at 10,000 g for 15 min, the supernatants were filtrated, and pH was checked (Přerovská et al., 2021). If needed, samples were further diluted to get within the calibration range of the following analysis.

The samples were analyzed using high-performance anion-exchange chromatography (HPAEC) with pulsed amperometric detection (PAD) system Dionex DX-600 (Dionex, United States) with anion-exchange column CarboPac PA1, 2 mm × 250 mm (Thermo Fisher Scientific, United States) for the possible presence of about 20 saccharides and sugar alcohols (modified method according to Hardy et al., 1988; Přerovská et al., 2021 and Nagel et al., 2014). The Dionex ECD-50 detector (Dow, United States) was switched to the PAD mode. The injection volume was 10 μl. The mobile phase flow rate was 0.25 ml∙min^−1^, and the column temperature was maintained at 25°C. The program starts at 0 min with a column in 100 mM NaOH, the NaOAc concentration is gradually increased to 240 mM during 50 min while maintaining the NaOH concentration at 100 mmol∙L^−1^. Then, within 0.5 min, there is a change to 100 mM NaOH/600 mM NaOAc and in such a way regeneration takes place until 55 min. Afterward, within 0.5 min, there is a smooth change to 200 mM NaOH regenerating the column until 58 min, and finally within 0.5 min there is another change to 100 mM NaOH causing reequilibration of the column until 65 min.

### *In vitro* Antifungal Assay

Antifungal activity of AGP-like enriched fraction and ulvan was measured according to the method previously described by Jindřichová et al. (2014). Briefly, GFP-tagged *L. maculans* (Šašek et al., 2012b) was suspended into 5 × 10^4^ spore∙ml^−1^ in a Gamborg B5 medium (Duchefa, Netherlands) supplemented with 0.3% sucrose and 10 mM MES pH 6.8. About 50 μl of conidia suspension was pipetted into black 96-well plate and then added 50 μl of test solutions (final concentration 0.01, 0.05, and 0.1 mg∙ml^−1^). AGP-like enriched fraction and ulvan were dissolved in 10 mM MES pH 6.8. As a growth control, 10 mM MES pH 6.8 was used. As positive control, 32 mM tebuconazole was used in form of commercial fungicide Horizon 250 EW (Bayer CropScience AG, Germany). The covered and micropore tape sealed plate was cultivated at 26°C and in the dark. Relative fluorescence was measured using Infinite F200 plate reader (TECAN, Switzerland) with filters for excitation 485/20 nm and for emission 535/25 nm every 24 h for 5 days. Fluorescent values were averaged for each treatment and difference between 96 and 0 h of control treatment was set as 100% of growth of *L. maculans*.

### Plant Treatment

Cotyledons of 12-day-old plants were used for AGP-like enriched fraction and ulvan solutions treatment. Lyophilized extracts of AGP-like enriched fraction and ulvan were dissolved in distilled water. For dissolving, solutions were slightly heated in water bath. As negative control treatment with distilled water was used and as positive control 32 μM benzothiadiazole (BTH), a synthetic analogue of salicylic acid, in the form of the commercial preparation Bion 50WG (Syngenta, Zambia) was used in induced resistance test. For all experiments, 12 plants were used for each treatment. Cotyledons were treated by infiltration using a syringe without needle until full leaf saturation. The final concentrations of AGP-like enriched fraction and ulvan were 0.01, 0.02, 0.05, and 0.1 mg∙ml^−1^.

### Induced Resistance Test

The 14-day-old plants were inoculated by conidia suspension of *L. maculans* in concentration 10^5^ spore∙ml^−1^. Inoculation was performed by infiltration using needleless syringe until complete leaf saturation. Infected leaves were evaluated by image analysis using the APS Asses 2.0 software (APS Press, United States). The lesion area relative to the cotyledon area was averaged for each treatment and compared to the control (water) treatment, representing 100%.

### Determination of Hydrogen Peroxide

Based on Thordal-Christensen et al. (1997), the presence of hydrogen peroxide was determined by the polymerization of 3,3′-diaminobenzidine (DAB). DAB solution (1 mg∙ml^−1^ in 10 mM Tris/HCl pH 7.8) was infiltrated into the cotyledons by vacuum infiltration. Infiltrated leaves were incubated for 4 h in dark at room temperature. Afterward, the chlorophyll was removed by several washes with 96% ethanol. Before scanning, the leaves were rehydrated by consecutive 75, 50, 25, and 0% ethanol washes. For longer storage were leaves kept in 50% glycerol. DAB forms a reddish–brown polymerization product in the presence of H_2_O_2_ and peroxidase (PX).

### Gene Transcription Analysis

RNA was isolated 24 h after plant treatment with studied compounds using commercial kit Spectrum™ Plant Total RNA Kit (Sigma Aldrich, United States). About 100 mg of plant material (10–12 disks with radius 6 mm) was used for isolation; four samples were collected from 12 plants. RNA was isolated according to the manufacturer manual and the concentration of isolated RNA was determined spectrophotometrically by NanoDrop 1000 (Thermo Scientific, United States). Isolated RNA (2.5 μg) was treated with DNA-free™ DNA Removal Kit (Ambion, United States) to remove possible contamination by genomic DNA. Isolated RNA was transcribed to cDNA *via* reverse transcription using M-MLV RNase H-point mutant (Promega, United States) and anchored oligo dT21 primer (Metabion, Germany). The qPCR reaction contained the equivalent of 6.25 ng of RNA in LightCycler® 480 SYBR Green I Master (Roche, Switzerland), in case of *ACS2* and *NCED3*, RNA equivalent was 25 ng. The final volume of reaction was 10 μl and was performed in a 96-well plate using LightCycler® 480 (Roche, Switzerland). The PCR conditions were 95°C for 10 min followed by 45 cycles of 95°C for 10 s, 55°C for 20 s, and 72°C for 20 s, followed by a melting curve analysis. Threshold cycles and melting curves were calculated using LightCycler®480 software. Level of relative transcription was calculated with an efficiency correction and normalized to the reference gene *Actin*. A list of primers is shown in Supplementary Table S1.

### Statistical Analysis

The experiments were carried out in three independent biological replicates (i.e., three separate experiments not conducted in parallel at the same time). Data were analyzed using pair *t*-test or one-way ANOVA with *post hoc* Tukey test (*p* < 0.05). All statistical analysis were performed using GraphPad Prism 8 software.

## Results

### Preparation of AGP-Like Enriched Fraction and Its Characterization

Based on physicochemical properties of both, AGPs and ulvan, ion-exchange (IEX) chromatography was chosen for their separation. In order to get rid of ulvan, purification procedure was optimized. The effectivity of separation was established based on the separation of AGP-like glycoproteins localized by western blot, and ulvan, whose localization was determined by TBO assay. The best results were achieved using Q Sepharose® Fast Flow resin and 25 mM MES buffer pH 6 and for the elution gradient of 2 M NaCl was chosen. The combination of step and linear elution gradient proved to be the most effective. Once the suitable protocol was found out, the purification was scaled up and the example chromatogram of chosen ion-exchange purification is presented in Supplementary Figure 1A.

The majority of proteins were localized within the peak containing AGP-like glycoproteins, represented by western blot positive fractions (Supplementary Figure S1B, lanes 6–8). On the other hand, the ulvan peak represented by TBO positive fractions contained almost no proteins (Supplementary Figure S1B, lane 9). The results of the western blot showed the presence of two high molecular weight AGP-like glycoproteins in JIM16 positive fractions. These fractions were collected and dialyzed against water for 3 days using a membrane with 100 kDa MWCO to desalt the sample and at the same time to remove low molecular weight compounds including the unwanted proteins. The dialyzed JIM16 positive fractions (AGP-like enriched fraction) were lyophilized afterwards and used for biological assays on plants.

Ulvan from *U. lactuca* was chosen as a control during the biological assays on plants, because of its well-documented elicitor activity. Ulvan from *U. lactuca* was prepared according to Yaich et al. (2013) and the yield was approximately 18% (w/w). To check the result of ulvan extraction, the sample was analyzed by FT-IR analysis (Supplementary Figure 2).

The measured FT-IR spectrum corresponded well to the already measured spectra of ulvan in the literature and contained all the bands typical for ulvan structure (Robic et al., 2009): the OH groups gave a signal at 3,420 cm^−1^, the uronic acids afforded expected signals at 1,634 and 1,428 cm^−1^, the sulfate groups absorbed at 1,258 and 1,225 cm^−1^, the glycosidic linkages absorbance band was at 1,138–1,127 cm^−1^, and the sugar-rings signals were assigned in the range of 110 and 990 cm^−1^ (Supplementary Figure 2B). However, some differences were noticed since the maximum absorption band at 1,135 cm^−1^ (1,055 cm^−1^ in Robic et al., 2009), and a shoulder between 1,220 and 1,130 cm^−1^ (not so significant in Robic et al., 2009) were observed. Although the ulvan extraction was successful, spotted differences might be pointing out to the slightly different structure of ulvan or presence of contamination within the sample. The presence of the bands at 656 and 645 cm^−1^ in the FT-IR spectrum suggested contamination by inorganic sulfates or phosphates.

Ulvan and AGP-like enriched fraction used for biological tests on plants were characterized mainly in terms of their glycosylation, which is assumed to be responsible for the AGPs functionality in plant development and defense responses (Lopez-Hernandez et al., 2020; Villa-Rivera et al., 2021).

**Figure 1:**
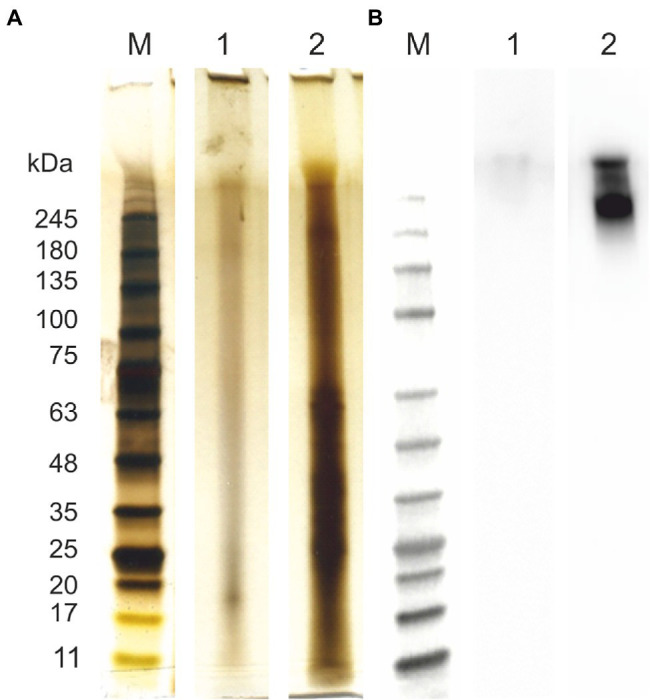
Protein characterization of arabinogalactan protein (AGP)-like enriched fraction (AGPs) and ulvan. Samples were analyzed using SDS-PAGE and silver staininig **(A)** and immunolabeling with anti-AGP JIM16 primary antibody, 10 s exposition time **(B)**. (1) Ulvan; (2) AGPs. For the SDS-PAGE separation were used 4%–15% gradient precast polyacrylamide gels. Prestained Protein Ladder—Broad molecular weight (10–245 kDa, ab116028) was used.

Firstly, all the samples were analyzed in terms of the total protein content and composition of AGP-like glycoproteins (Figure 1A). Even though almost no proteins and mainly smear typical for polysaccharides could be seen in the case of the extracted ulvan (Figure 1A; lane 1), the presence of proteins confirms the contamination of extracted ulvan. The AGP-like enriched fraction contained a high amount of proteins with a molecular weight below 75 kDa (Figure 1A; lane 2). Surprisingly, even though the majority of the unwanted proteins had molecular weight below 75 kDa, they were not removed by 3-day 100 kDa MWCO dialysis at all. Nevertheless, the JIM16 antibody had a strong response with the sample after IEX purification (Figure 1B; lane 2). Besides, an almost invisible response could be seen also in extracted ulvan (Figure 1B; lane 1). These findings further correspond to their spectrophotometric analysis of protein, total saccharide, and uronic acid content (Table 1).

**Table 1:** The characterisation of samples.

A—Total content			
	AGPs	Ulvan	
Protein	0.346 ± 0.027	0.041 ± 0.004	
Total saccharides	0.191 ± 0.027	0.276 ± 0.037	
Uronic acids	0.082 ± 0.002	0.200 ± 0.010	
**B—Neutral monosaccharide composition**			
**Retention time (min)**	**AGPs**	**Ulvan**	
2.93	1.4 ± 0.3	Traces	Fucose
3.15	27.7 ± 2.3	6.8 ± 0.5	3-*O*-methyl-hexose^a^
3.42	15.7 ± 0.5	71.5 ± 2.8	Rhamnose
3.92	12.5 ± 0.3	n.d.	-^b^
4.13	10.0 ± 0.8	Traces	Arabinose
4.63	15.5 ± 0.1	0.8 ± 0.1	-^b^
4.83	4.5 ± 0.1	2.3 ± 0.2	Mannose
5.05	7.3 ± 0.1	9.8 ± 0.2	Galactose
5.07	1.8 ± 0.1	traces	Glucose
5.27	3.8 ± 0.1	8.0 ± 0.5	Xylose
**C—Negatively charged monosaccharide composition**			
**Retention time (min)**	**AGPs**	**Ulvan**	
25.15	13.9 ± 0.9	n.d.	-^c^
25.83	23.4 ± 0.3	n.d.	Galacturonic acid
27.65	30.7 ± 0.8	33.4 ± 1.3	Glucuronic acid
33.90	3.7 ± 0.1	6.4 ± 0.2	Iduronic acid
36.47	28.4 ± 0.2	60.2 ± 1.9	-^c^

**(A)** Basic characterization of the samples by spectrophotometric methods. Protein, total saccharide, and uronic acid mass fraction of Ulva lactuca ulvan, and AGP-like enriched fraction. Protein content was measured by the Bicinchoninic Acid (BCA) method, total saccharide content by anthrone method, and uronic acid content by the 3-hydroxybiphenyl method. Values in the table represent the mean ± SE (*n* = 9, collected from three independent experiments). **(B)** Neutral monosaccharide composition (mass % of total neutral saccharides) of Ulva lactuca ulvan, and AGP-like enriched fraction. Values in the table represent the mean ± SE (*n* = 9, collected from three independent experiments). **(C)** Negatively charged monosaccharide composition (mass % of total negatively charged monosaccharides) of Ulva lactuca ulvan, and AGP-like enriched fraction. Values in the table represent the mean ± SE (*n* = 4, collected from two independent experiments).

a Approximate content, the concentration calculated with a coefficient of 3-*O*-methyl-glucose.

b Approximate content, the concentration calculated with average coefficient of all standards.

c Approximate content, the concentration calculated with an average coefficient of all uronic acid standards.

Traces: content < 0.5%; n.d., not detected.

The neutral saccharide composition differed greatly between the samples (Table 1B). Although the ulvan composition corresponded to the information present in the literature, surprising was the presence of unidentified monosaccharides (Yaich et al., 2013). One of these unidentified monosaccharides (retention time 3.15 min) was previously identified as 3-*O*-methyl-hexose, possibly 3-*O*-methyl-galactose, which has never been described in ulvan structure and might originate from the contaminating proteins (Přerovská et al., 2021). This saccharide was the most prevalent saccharide within AGP-like enriched fraction, followed by rhamnose, saccharide with retention time 4.63 min, saccharide with retention time min 3.92 min, and arabinose. The content of the remaining saccharides did not exceed 10%. Interestingly, the saccharide with retention time 3.92 min could be found only in this sample.

Moreover, the composition of negatively charged monosaccharides was completely different too (Table 1C). Ulvan negatively charged monosaccharide composition is almost identical to the *Ulva* extract with the majority of negatively charged monosaccharide with retention time 36.47 min. The data for *Ulva* extract were previously published in Přerovská et al. (2021). After IEX purification of *Ulva* extract the amount of this unidentified negatively charged monosaccharide, glucuronic, and iduronic acid decreased, whereas a significant amount of negatively charged monosaccharide with retention time 25.15 min and galacturonic acid appeared in the sample.

For the simplification, the term AGP-like enriched fraction will be in following text shortened to AGPs. It is a mixture of AGP-like glycoproteins and other proteins. However, low molecular weight compounds such as phytohormones and the vast majority of ulvans were removed from the sample during preparation. It is important to keep in mind that the structure and composition of *U. lactuca* AGP-like glycoproteins differ significantly from the AGPs of classical terrestrial plants (Seifert and Roberts, 2007; Přerovská et al., 2021).

### AGP-Like Enriched Fraction Protects *Brassica napus* Against *Leptosphaeria maculans*

**Figure 2:**
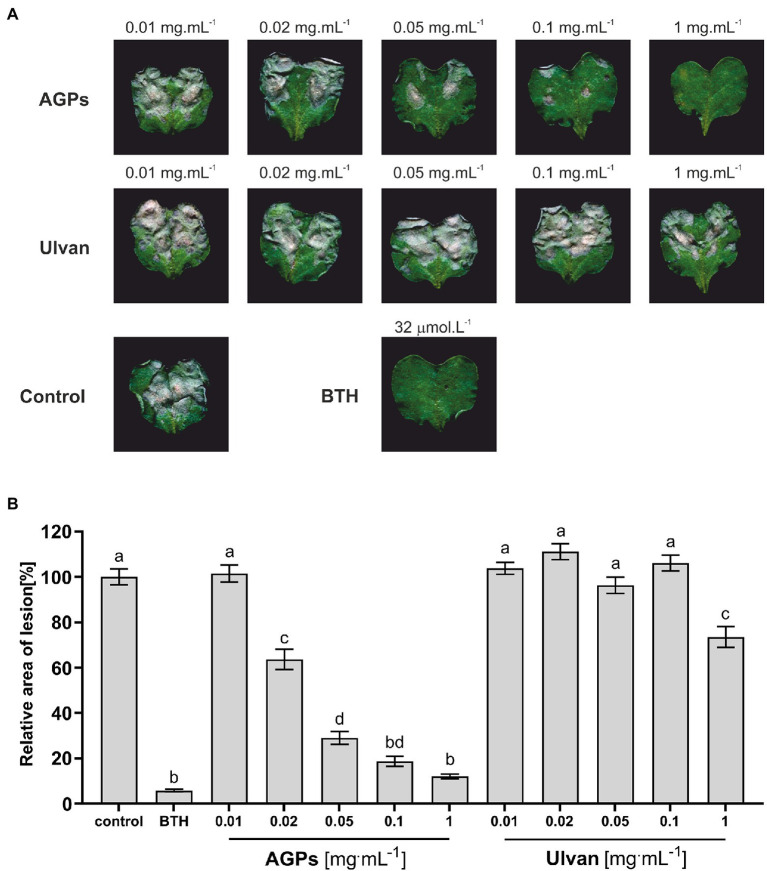
Effect of algal elicitors on the progression of *L. maculans* infection in *B. napus* cotyledons. Cotyledons were treated with AGP-like enriched fraction (AGPs), ulvan extracted according to Yaich et al. (2013) (Ulvan), distilled water (negative control), and 32 μM BTH (benzothiadiazole, positive control) 2 days before inoculation with *L. maculans*. Symptoms of *L. maculans* infection on cotyledons of *B. napus* 11 days after inoculation **(A)**. Disease symptoms were evaluated as a percentage of the lesion area to the leaf area 11 days after inoculation **(B)**. The algal elicitors were tested in concentrations 0.01, 0.02, 0.05, 0.1, and 1 mg·ml^−1^. The graph presented data from three biological replicates. Statistically significant differences determined by the one-way ANOVA and Tukey *post-hoc* test (*p* < 0.05). Each column is presented as the mean ± SE (*n* = 72). Different letters indicate significant difference.

The protection efficacy of five different concentrations of algal elicitors (AGPs or ulvan) in *B. napus* against *L. maculans* was assessed by infiltration of *B. napus* cotyledons 2 days prior to inoculation with the pathogen. Once the lesions have developed (11 days after inoculation), the cotyledons were scanned to evaluate the lesion area (Figure 2A).

The grey-brown areas represent the *L. maculans* lesions. From the images themselves it was obvious, that AGPs caused a significant reduction in disease progression in concentration-dependent manner with concentration 1 mg·ml^−1^ being as effective as 32 μM benzothiadiazole (BTH), which was used as a positive control. BTH is a synthetic analog of salicylic acid able to induce SA-mediated stress response, which plays a major role in the defense against hemibiotrophic pathogens. Moreover, the lesion area was evaluated by image analysis, when the lesion area relative to the cotyledon area was averaged for each treatment and compared to the control treatment, expressed as 100%. Each treatment was represented by 12 plants and the whole experiment was repeated three times (Figure 2B).

The concentration-dependent effect of AGPs on the reduction of *L. maculans* infection is even more profound from the graph (Figure 2B). As positive control was used treatment with BTH, which diminished infection propagation by 99%–92% compared to control plants. Even the second-lowest tested concentration (0.02 mg·ml^−1^) led to a decrease in the relative area of lesions by 45%–30%. The pretreatment with the highest tested concentration (1 mg·ml^−1^) resulted in a major reduction of infection propagation by 94%–83%, which was almost as efficient as the use of commercial elicitor BTH. Unexpectedly, the ulvan pretreatment had much lower elicitor activity, when only the highest tested concentration caused a statistically significant drop in lesion relative area by 47%–15%. Besides, greater variability between individual biological repetitions could be observed in the case of ulvan results, especially at higher concentrations.

### AGP-Like Enriched Fraction Did Not Display Any Direct Antifungal Activity Against *Leptosphaeria maculans*

**Figure 3:**
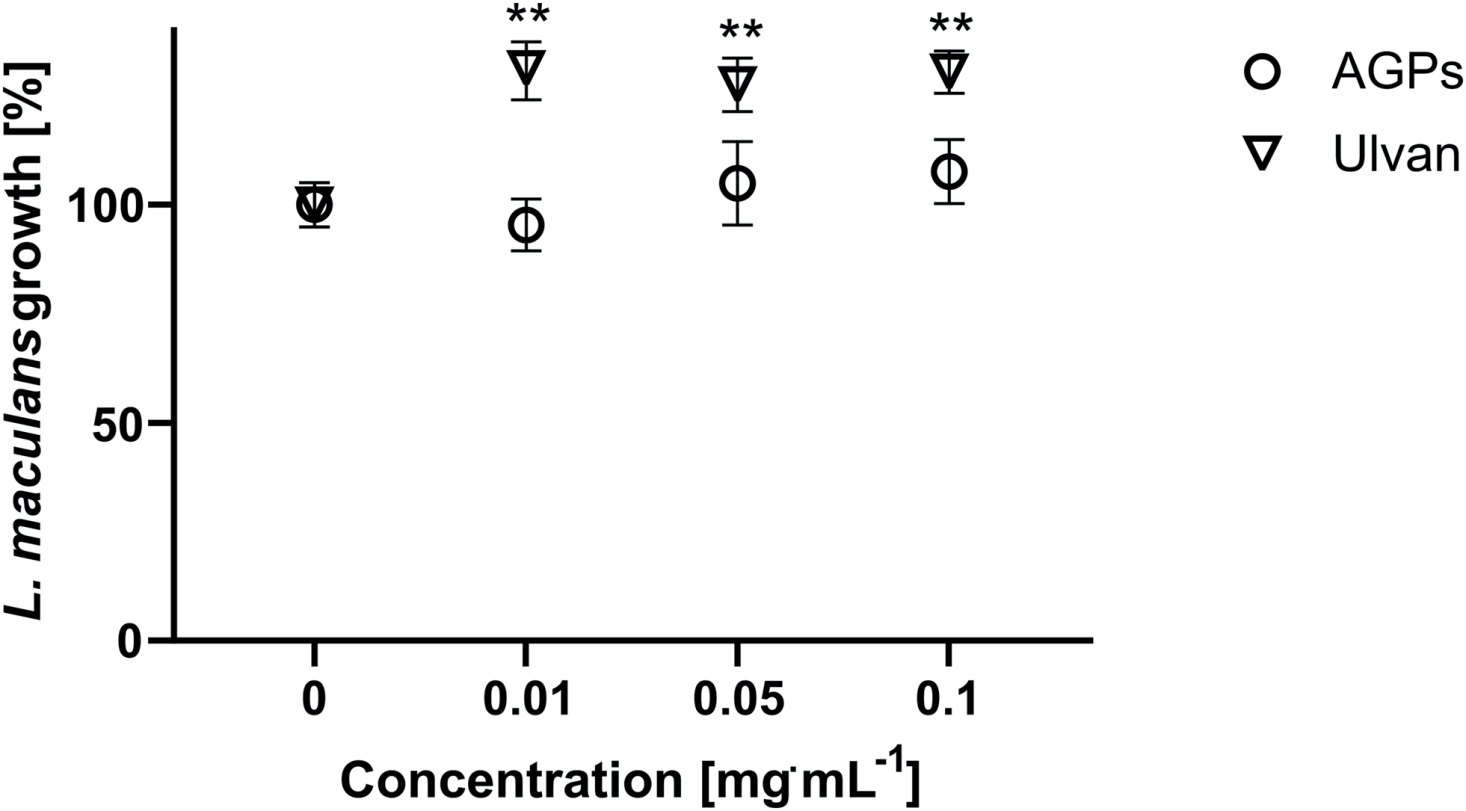
Antifungal effect of AGP-like enriched fraction (AGPs) and ulvan on *L. maculans* growth. Spores of *L. maculans* tagged with GFP were cultivated with different concentrations of AGPs and ulvan extracted according to Yaich et al. (2013) (Ulvan) for 96 h in a microtitre plate. The growth of mycelium was quantified as an increase in GFP fluorescence. The data are presented as the mean ± SE values (*n* = 6). Statistically significant differences determined by the *t*-test are marked either with an ^*^p < 0.05 or ^**^p < 0.01, all samples were compared to the control (10 mM MES pH 6.8).

To exclude a direct antifungal effect of the tested compounds, the direct antifungal effect of AGPs and ulvan on *L. maculans* was examined *in vitro*. The assay showed that the relative fluorescence of growing mycelium of *L. maculans* did not significantly differ among the control and AGPs. Interestingly, ulvan in all tested concentrations improved *L. maculans* growth (Figure 3).

### AGP-Like Enriched Fraction Induced Production of H_2_O_2_ in *Brassica napus* Cotyledons

Hydrogen peroxide represents important ROS, which has been shown to participate in cell signaling regulation, differentiation, programmed cell death, cell wall formation, and stress responses to both abiotic and biotic factors (Huang et al., 2019).

**Figure 4:**
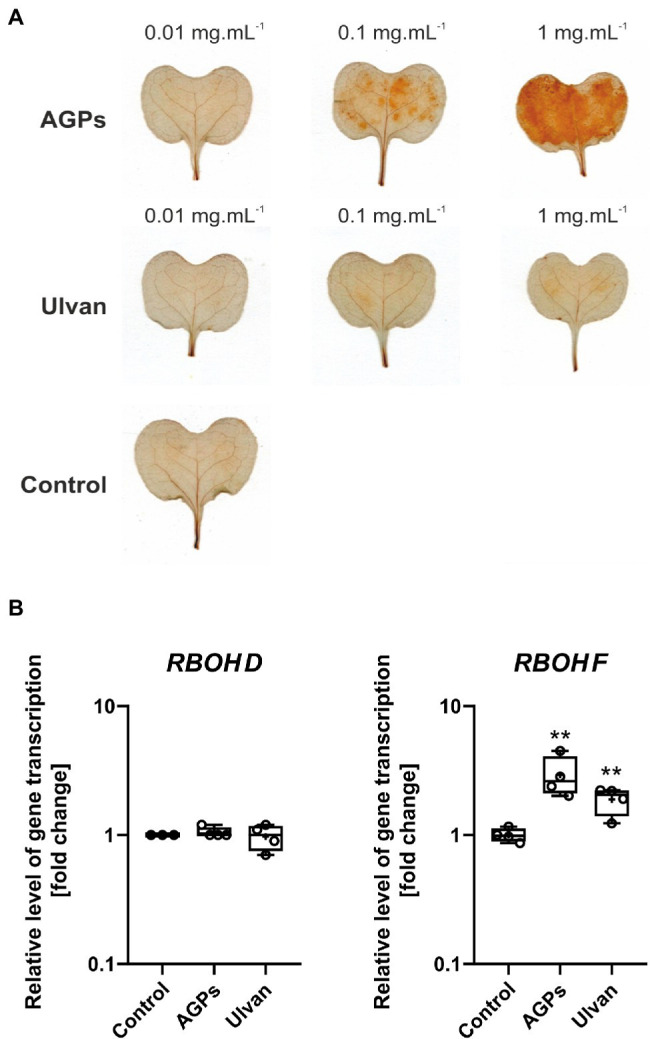
Effect of algal elicitor treatment on accumulation of H_2_O_2_ and expression of *respiratory burst oxidase homologues* (*RBOHs*) in *B. napus* cotyledons. Accumulation of H_2_O_2_ in *B. napus* cotyledons **(A)**. Cotyledons (12-day-old) were treated with AGP-like enriched fraction (AGPs), ulvan extracted according to Yaich et al. (2013) (Ulvan), and distilled water (Control) by infiltration, the algal elicitors were tested in concentrations 0.01, 0.1, and 1 mg·ml^−1^. H_2_O_2_ was detected 24 h after treatment using 3,3′-diaminobenzidine, the presence of H_2_O_2_ is represented by brown-red coloring. Expression of *RBOHs* in *B. napus* cotyledons **(B)**. Cotyledons were treated with AGP-like enriched fraction (AGPs), ulvan (Ulvan), and distilled water (Control) by infiltration in 0.1 mg·ml^−1^ concentration. After 24 h, gene expressions of *RBOH D* and *RBOH F* were analyzed. Data from the representative experiment are shown. Relative expression was calculated with efficiency correction and normalization to *actin*. Data are plotted at the log_10_ scale. Asterisks indicate statistically significant differences from control determined by the *t*-test ^**^p < 0.01.

The formation of ROS is the first defense response of plants to biotic and abiotic stress and was suggested to play a pivotal role in the establishment of SAR with H_2_O_2_ as intra- and intercellular messenger (Barna et al., 2012). Thus, the effect of AGPs from *U. lactuca* and extracted ulvan on the formation of H_2_O_2_ was examined (Figure 4A).

The infiltration of AGPs sample into the cotyledons of *B. napus* leads to the accumulation of H_2_O_2_ in a concentration-dependent manner. Only weak accumulation of H_2_O_2_ was detected after treatment with ulvan regardless of the concentration used. In the case of water infiltration, H_2_O_2_ accumulation was not observed. To determine the origin of produced hydrogen peroxide, relative gene expression of two NADPH oxidases, also called respiratory burst oxidase homologues (RBOHs) was assessed (Figure 4B). Although RBOH family has more members, RBOH D and RBOH F are believed to be the key players in the ROS production during the stress responses (Chapman et al., 2019).

Treatment of *B. napus* cotyledons with AGPs and ulvan 24 h prior measurement resulted in increased expression of *RBOH F* by 2.9- and 1.9-fold compared to control, respectively. In the case of *RBOH D*, the expression level remained unchanged.

### AGP-Like Enriched Fraction Induced Expression of Plant Defense Genes

**Figure 5:**
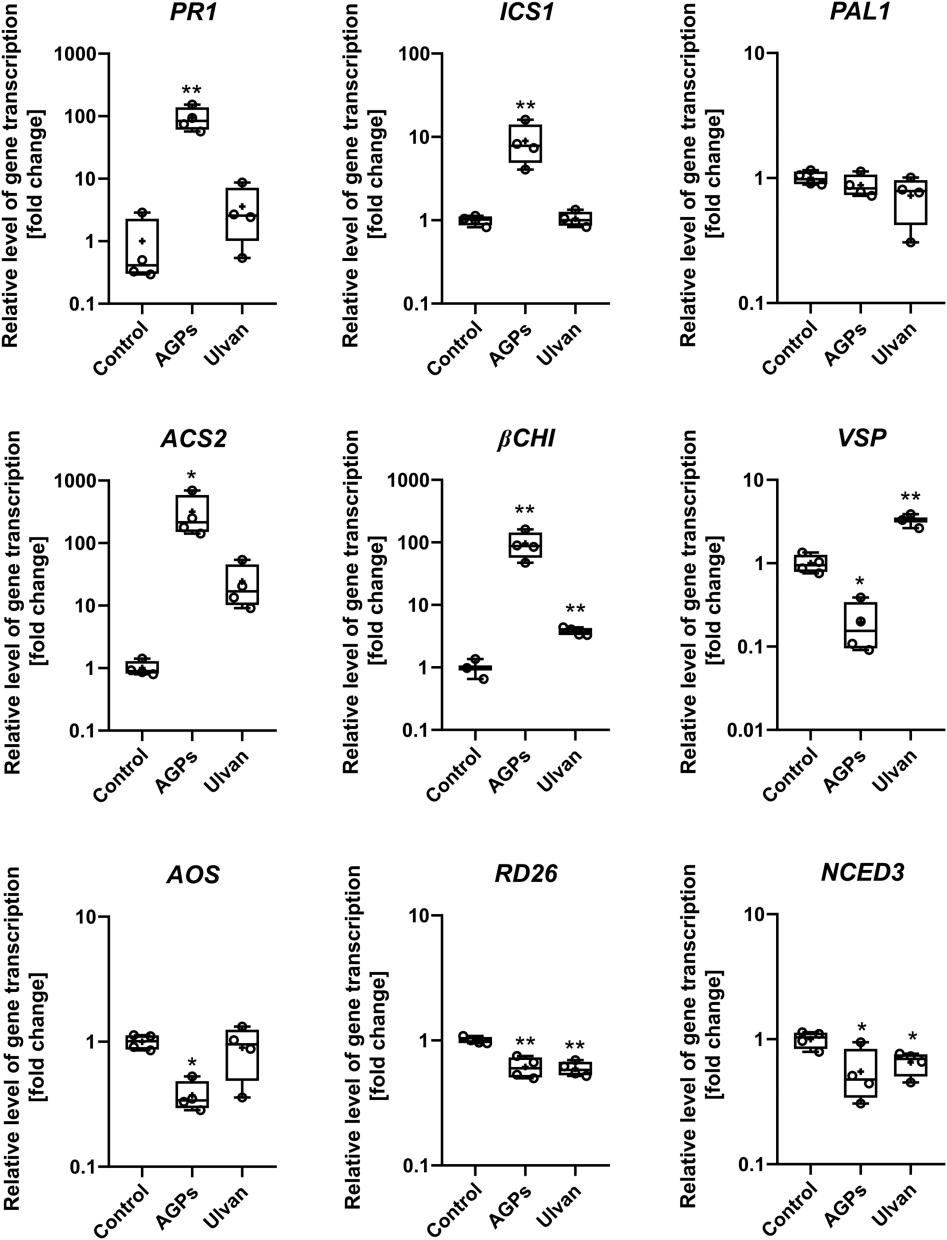
Effect of algal elicitor treatment on activation of plant defense pathways in *B. napus* cotyledons. Cotyledons were treated with AGP-like enriched fraction (AGPs), ulvan extracted according to Yaich et al. (2013) (Ulvan) and distilled water (Control) by infiltration in 0.1 mg·mL^−1^ concentration. After 24 h, gene expressions of marker genes of salicylic acid pathway (*PR1*, *ICS1*, and *PAL1*), ethylene pathway (*ACS2*), ethylene/jasmonic acid pathway (*βCHI*), jasmonic acid (*VSP* and *AOS*), and abscisic acid (*RD26*, *NCED3*) were analyzed. Data from the representative experiment are shown. Relative expression was calculated with efficiency correction and normalization to *actin*. Data are plotted at the log_10_ scale. Asterisks indicate statistically significant differences from control determined by the *t*-test ^*^p < 0.05 or ^**^p < 0.01.

To further understand the mechanisms behind improved *B. napus* resistance to *L. maculans* infection, the effect of AGPs on the activation of signaling pathways was tested and compared to control and ulvan treatment. Marker genes linked to the individual signaling pathways were chosen and their changes in expression 24 h after elicitor infiltration were observed. Corresponding to the previous results of inoculation assay, the treatment with AGPs caused statistically significant changes of gene expression in the *B. napus* cotyledons (Figure 5).

The relative expression of the nine genes involved in *B. napus* defense reactions—namely, *pathogenesis-related gene 1* (*PR1*), *isochorismate synthase 1* (*ICS1*), *phenylalanine ammonia-lyase* (*PAL*), *ACC synthase* (*ACS2*), *β-chitinase* (*βCHI*), *vegetative storage protein* (*VSP*), *allene oxide synthase* (*AOS*), transcription factor *responsive to desiccation 26* (*RD26*), and *9-cis-epoxycarotenoid dioxygenase 3* (*NCED3*)—were analyzed using RT-qPCR in water- (Control), AGPs-, and ulvan-treated plants. The treatment with AGPs led to the activation of the salicylic acid signaling pathway based on increased expression of SA responsive gene *PR1* (94.4-fold) as well as SA biosynthetic gene *ICS1* (8.9-fold). Though, the biggest change in gene expression could be observed in the case of *ACS2* (316.6-fold), pointing to strong activation of ethylene signaling pathway. Although the elevated level of *βCHI* expression (95.9-fold) indicates the involvement of the JA/ET signaling pathway, marker genes for jasmonic acid pathway *AOS* and *VSP* were downregulated by 0.4- and 0.2-fold, respectively. On contrary, ulvan in addition to the upregulation of *βCHI* (3.8-fold) also increased expression of *VSP* (2.6-fold), suggesting activation of JA/ET signaling pathway. Moreover, marker genes of abscisic acid pathway, *RD26* and *NCED3*, were downregulated by both AGPs (0.6- and 0.6-fold) and ulvan (0.6- and 0.7-fold). The changes in expression of the other marker genes were not statistically significant. The results suggest an unusual synergistic role of SA and ET signaling pathways during the AGPs induced resistance.

## Discussion

Seaweed extracts are already used in agriculture for their growth-promoting activity and ability to enhance plant stress tolerance for decades (Battacharyya et al., 2015). Although *Ascophyllum nodosum* products are the most commercially used (Sharma et al., 2014), *Ulva* sp. extracts have also high potential and have been the topic of various research papers (Dominguez and Loret, 2019). The most crucial challenge in seaweed biostimulant development is to choose the right extraction protocol, which will harvest all desired molecules with biostimulant activity. The protocol immensely affects the composition of final product and various protocols were established over the years. Although novel extraction technologies such as supercritical fluid extraction or microwave-assisted extraction are available, at the industrial level, the most common method is heating of algal biomass with potassium or sodium hydroxide solutions under pressure. Such harsh conditions can lead to the loss of some bioactive compounds plus result in uncontrolled fragmentation of polysaccharide chains, which consequently affect the biostimulant activity of the formulation (El Boukhari et al., 2020; Ali et al., 2021). Our results show, that even mild buffer extraction yields high amounts of proteins and saccharides with biostimulant activity within our extract. The eliciting activity of seaweed extracts is mainly attributed to the sulfated polysaccharides presented in their cell walls (Stadnik and Freitas, 2014). Nevertheless, algae contain a tremendous number of other biomolecules with potential biostimulant activity and AGPs belong to them. AGPs play a crucial role in higher plant defense responses and plant-microbe interactions (Nguema-Ona et al., 2013; Mareri et al., 2019), and their presence was confirmed also in brown and green algae (Estevez et al., 2009; Hervé et al., 2015; Ma et al., 2017; Přerovská et al., 2021). To test the ability of *U. lactuca* AGP-like glycoproteins to elicit defense responses and enhance stress resistance of higher plants, an AGP-like enriched sample containing approximately one-third of AGP-like glycoproteins was prepared by IEX chromatography. The ulvan extracted from *U. lactuca* was used for comparison. It activates plant immunity through the RBOH-dependent JA signaling pathway without inducing hypersensitive response (HR; Jaulneau et al., 2010; Freitas and Stadnik, 2015; Martin et al., 2020).

Up to now, seven studies focusing on the effects of different *Ulva* spp. extracts on plant infections with pathogens were published. Most of the studies used identical extraction protocol based on Cluzet et al. (2004), where dried *Ulva* material was autoclaved in distilled water, followed by ethanol precipitation. Some of the studies then call their product ulvan or some more generally as *Ulva* extract. In this study, ulvan was prepared according to Yaich et al. (2013) and based on excellent review of ulvan extractions done by Kidgell et al. (2019); our protocol should have high extraction yield, selectivity and low degradation. Only six studies also tried to analyze their products, mainly by means of spectrophotometric analyses, monosaccharide composition, and FT-IR analyses (Cluzet et al., 2004; Paulert et al., 2009, 2010; Jaulneau et al., 2011; Hernández-Herrera et al., 2014; Borba et al., 2019). Ulvan is mainly composed of rhamnose and glucuronic acid with the main repeating disaccharide unit (→4)-β-d-GlcA*p*-(1 → 4)-α-l-Rha*p*-(1→, in which glucuronic acid can be replaced to a certain extent by iduronic acid or xylose). Sulfation occurs mainly on C3 of the rhamnose and also C2 of the xylose or glucuronic acid (Lahaye and Robic, 2007; Kidgell et al., 2019). Although our results of ulvan analyses agrees with previously published data, outstanding is the presence of 3-*O*-methyl-hexose and unidentified saccharides, which possibly comes from contaminating glycoproteins. The most intriguing is the nature of negatively charged monosaccharide with retention time 36.47 min. Nevertheless, based on the knowledge of ulvan composition and the separation principle of the HPAEC/PAD technique, its long elution time indicates a strongly polar nature suggesting that we are dealing with a sulfated monosaccharide such as rhamnose-3-sulfate, which would correspond to its high content in ulvan (Templeton et al., 2012; Yaich et al., 2013). The composition of AGP-like enriched samples differs greatly from ulvan containing greater variety of monosaccharides and even more unidentified ones including rhamnose-3-sulfate. If true, the origin of this sulfated monosaccharide in the sample purified by ion-exchange chromatography, which should not contain ulvan, remains unknown. However, the correlation between sulfation and salt tolerance was previously proven (Aquino et al., 2011). Thus, the hypothetical presence of sulfated monosaccharide within AGP-like glycoproteins might be an adaptation to the marine environment. The other unidentified negatively charged monosaccharide with retention time 25.15 min is most probably 4-*O*-methyl-glucuronic acid, which would be in agreement with Pfeifer et al. (2020), who found out that *Zostera marina* AGPs contained high amounts of glucuronic acid and terminal 4-*O*-methyl-glucuronic acids, rare to land plant AGPs. We hypothesize, that the presence of unusual, modified monosaccharides might play an important role in eliciting plant resistance against the pathogen. The presence of galacturonic acid is interesting since it has rarely been described as part of AGP glycans (Tan et al., 2013). Though, high content of galacturonic acid and glucuronic acid was also identified in AGP-like glycoproteins of *Micrasterias denticulata* (Eder et al., 2008), which pointed out to the unique glycosylation of algal AGP-like glycoproteins. The high content of uronic acids was proposed as a specific adaptation to the marine environment, thanks to their calcium-binding capacity and the ability of calcium ions to protect plants from harmful effects of salt stress (Lahaye and Epstein, 1969, 1971; Cramer et al., 1985; Pfeifer et al., 2020). The ion-binding capacity of AGPs can be fine-tuned according to environmental factors (Lamport and Várnai, 2013; Pfeifer et al., 2020). Moreover, the essential role of pH-dependent periplasmic AGP–Ca^2+^ capacitor in signaling and normal plant development was reported (Lamport and Várnai, 2013; Lamport et al., 2014, 2018; Mizukami et al., 2016; Lopez-Hernandez et al., 2020). Importantly, big differences in composition can be found between the AGP-like enriched fraction prepared with the help of IEX chromatography and AGP-like glycoproteins obtained by Yariv precipitation from *U. lactuca* extract, whose composition was previously published (Přerovská et al., 2021). The discrepancies are caused most probably due to the distinct content of individual AGP-like glycoproteins, or the presence of contaminating compounds based on different purifications. For instance, the IEX purified product contained more proteins other than AGP-like glycoproteins, and possibly a large fraction of them is glycosylated making the data difficult to interpret. Moreover, Yariv precipitated AGP-like glycoproteins are highly enriched in AGP-like glycoprotein with molecular weight approximately 20 kDa compared to AGP-like enriched fraction prepared by IEX chromatography. Nevertheless, the composition of Yariv precipitated AGP-like glycoproteins still differed greatly from data known from land plant AGPs. Most importantly, the use of Yariv reagent is not suitable for large scale purification, due to the cost of Yariv reagent and mainly due to extremely low yields of the purification (Přerovská et al., 2021). Our results showed that pretreatment of *B. napus* plants with AGPs significantly reduced the development of *L. maculans* symptoms on cotyledons. To reveal the mechanism of fungus retardation induced by AGPs treatment, the direct antimicrobial activity tests of the compounds were performed in axenic cultures *in vitro*. Neither AGPs nor ulvan had any effect on the growth of *L. maculans* in this study. Unfortunately, no similar data are available for comparison. However, in the case of ulvan, various data can be found showing either no direct antifungal effect toward different pathogens (Freitas and Stadnik, 2012) or even enhancing the germination of conidia of *C. lindemuthianum* (Paulert et al., 2009). The latter corresponds to the enhanced growth of *L. maculans*, which can be explained in the same way. Simply, the polysaccharide can serve as a carbon source for the fungus. Nevertheless, since the compounds studied did not show any direct antifungal effect, but at the same time were able to reduce the severity of *L. maculans* infection at a certain concentration, it can be assumed that the protection is due to their elicitor activity. In general, elicitors trigger numerous signaling events that lead to the activation of the defense. Among the earliest is the ROS production of superoxide, hydroxyl radical and hydrogen peroxide. The latter plays a central role in biotic stress, including oxidative burst, cross-linking of cell wall proteins, callose deposition, signaling, defense gene expression, and hypersensitive response often manifested by systemic acquired resistance (Freitas and Stadnik, 2015; Waszczak et al., 2018). While AGPs caused a concentration-dependent production of H_2_O_2_, almost no H_2_O_2_ was produced after treatment with ulvan. Similar H_2_O_2_ accumulation was also found in *B. napus* cotyledons infiltrated with an oligosaccharide elicitor isolated from *L. maculans* mycelium (Kim et al., 2013).

These results are consistent with gene expression analysis, as ROS can potentiate the production of SA and SA-mediated signaling, leading to the expression of SA-responsive defense genes such as *PR1*. These findings agree with the proposed mode of action of *A. nodosum* extract (Stella Maris®; Cook et al., 2018). Moreover, the H_2_O_2_ produced could likely have direct antimicrobial activity, as the inhibitory effect of hydrogen peroxide on conidial germination and mycelial growth of *L. maculans* (Jindřichová et al., 2011) has been described previously. It is noteworthy that H_2_O_2_ production could be partly caused by phytotoxicity of AGPs, as necrosis formed after treatment with a high concentration (10 mg·ml^−1^, data not shown). However, this fact is not a problem as even low concentrations lead to a significant reduction in the severity of infection without phytotoxic effects. Phytotoxicity phenomenon has already been described for other elicitors (Burketova et al., 2015; Trdá et al., 2019). Several articles describing the effect of ulvan treatment on H_2_O_2_ production showed a different response depending on the plants used and the priming of ROS production (Paulert et al., 2010; Abouraïcha et al., 2015; Freitas and Stadnik, 2015). Although our results seem to contradict the findings of Freitas and Stadnik (2015), where ulvan treatment resulted in higher increase of H_2_O_2_ production in *A. thaliana* compared to our findings, they used ulvan from *Ulva fasciata* and a different extraction methodology. Ulvan composition is highly dependent on the source species, ecophysiology, extraction, and processing procedure, which causes diverse bioactivity profiles (Kidgell et al., 2019). In order to determine the origin of the hydrogen peroxide produced, the expression of two NADPH oxidases, also called respiratory burst oxidase homologs (RBOHs), was analyzed. *RBOH D* and *RBOH F* were chosen, since they are known to be key players in stress responses in various plant pathosystems (Torres et al., 2002; Morales et al., 2016; Jasso-Robles et al., 2020). Both AGPs and ulvan caused a significant increase in the expression of *RBOH F*, but not *RBOH D*. Although these two enzymes cooperate during ROS generation, they are thought to play different roles in the regulation of hypersensitive response. While *RBOH D* is responsible for most of the ROS production during effector-triggered immunity, *RBOH F* is thought to control cell death (Torres et al., 2002). Moreover, a different expression pattern of these two NADPH oxidases has been demonstrated, with *RBOH F* being mainly expressed in leaves. Nevertheless, striking differences between their functions are evident in the literature depending on the pathosystem studied and even on the inoculation method, plant growth conditions or sampling time (Morales et al., 2016). Moreover, NADPH oxidases are not the only sources of hydrogen peroxide during defense responses. Polyamine oxidases and cell wall peroxidases also contribute (Kámán-Tóth et al., 2019; Jasso-Robles et al., 2020).

In addition to H_2_O_2_ accumulation, the treatment of plants with AGPs caused changes in defense genes transcription. The results suggest that increased resistance of *B. napus* against *L. maculans* elicited by AGPs is SA-dependent as indicated by elevated transcription of both SA-biosynthetic gene *ICS1* and SA-responsive gene *PR1*. Since the expression of *PAL* did not differ from the control, it is probable that SA is synthesized exclusively *via* the pathway regulated by *ICS1*. In addition to the salicylic acid signaling pathway, the AGPs induced also expression of *βCHI* gene involved in JA/ET signaling. On the other hand, the activation of *AOS* transcription, the biosynthetic gene for jasmonic acid, by AGPs, was not observed. This resembles the signaling situation reported by Šašek et al. (2012b) within *B. napus* infection with *L. maculans* who showed, that the main signaling pathways involved in this pathosystem are SA and ET signaling and that the transcription of the related genes was significantly increased 7 days after pathogen recognition. This is further supported by the strong transcription of *ACS2*, the ethylene-biosynthetic gene, elicited by AGPs treatment. Our findings indicate elicitation of both SA-dependent and ET-dependent signaling pathways. Nevertheless, the ever-increasing discoveries of crosstalks in between the signaling pathways revealed the truly complex nature of plant responses (Bürger and Chory, 2019; Yang et al., 2019), even in the plant *B. napus* (Nováková et al., 2014). Besides our results correspond to the results of Cluzet et al. (2004), who described the increased expression of *CHI*, *PR1*, and *PR10* genes after treatments with various *Ulva* extracts. The activation of *PR1* transcription was also reported in *B. napus* cotyledons after treatment with oligosaccharide elicitor isolated from *L. maculans* mycelium (Kim et al., 2013) and protein elicitor isolated from *L. maculans* cultivation medium (Nováková et al., 2016). The high production of hydrogen peroxide together with a strong induction of SA and ET signaling pathways explains significant inhibitory effects of AGPs on the infection development.

Surprisingly, ulvan caused almost no significant changes in gene expression except for slightly increased levels of *βCHI* and *VSP*, consistent with its known mode of action (Jaulneau et al., 2010; Hernández-Herrera et al., 2016; Ramkissoon et al., 2017). Based on the results, the *U. lactuca* ulvan appeared to be efficient in the pathosystems studied at higher concentrations, which was further supported by an 80% decrease in *L. maculans* infection after treatment with a concentration of 10 mg·ml^−1^ (Supplementary Figure 3). The variability in ulvan data may be due to the viscous nature of the concentrated samples, which causes uneven infiltration.

In addition to the major defense signaling pathways regulated by SA, JA, and ET, *B. napus* plants responded to elicitor treatment with a decrease in genes related to ABA. Both the *NCED3* biosynthetic gene and the *RD26* responsive gene were downregulated by both the AGPs and ulvan. This result is in accordance with previous findings of Jaulneau et al. (2010), who reported a transient decrease (2 days after treatment) in ABA-responsive genes in *Medicago truncatula*. On the other hand, Chen et al. (2013) found an increase in ABA in plants treated with a protein elicitor from oomycete *Phytophthora boehmeriae*, which lead to significant reduction of pathogen infection. These contrasting results indicate that the role of ABA in induced resistance by elicitors is not as straightforward as, e.g., the role of SA. The role of ABA in plant defense against pathogens is less defined and the data are less consistent compared with SA, JA, and ET signaling. Since ABA regulates stomata opening, it is suggested that ABA is an important phytohormone in protecting the host plant from pathogen penetration *via* the stomata. The possible positive role of ABA in the studied pathosystem *B. napus—L. maculans* was previously reported by Šašek et al. (2012b). Similar to ABA, several elicitors of different origins induced stomata closure and ROS production in guard cells (Allègre et al., 2009).

**Figure 6:**
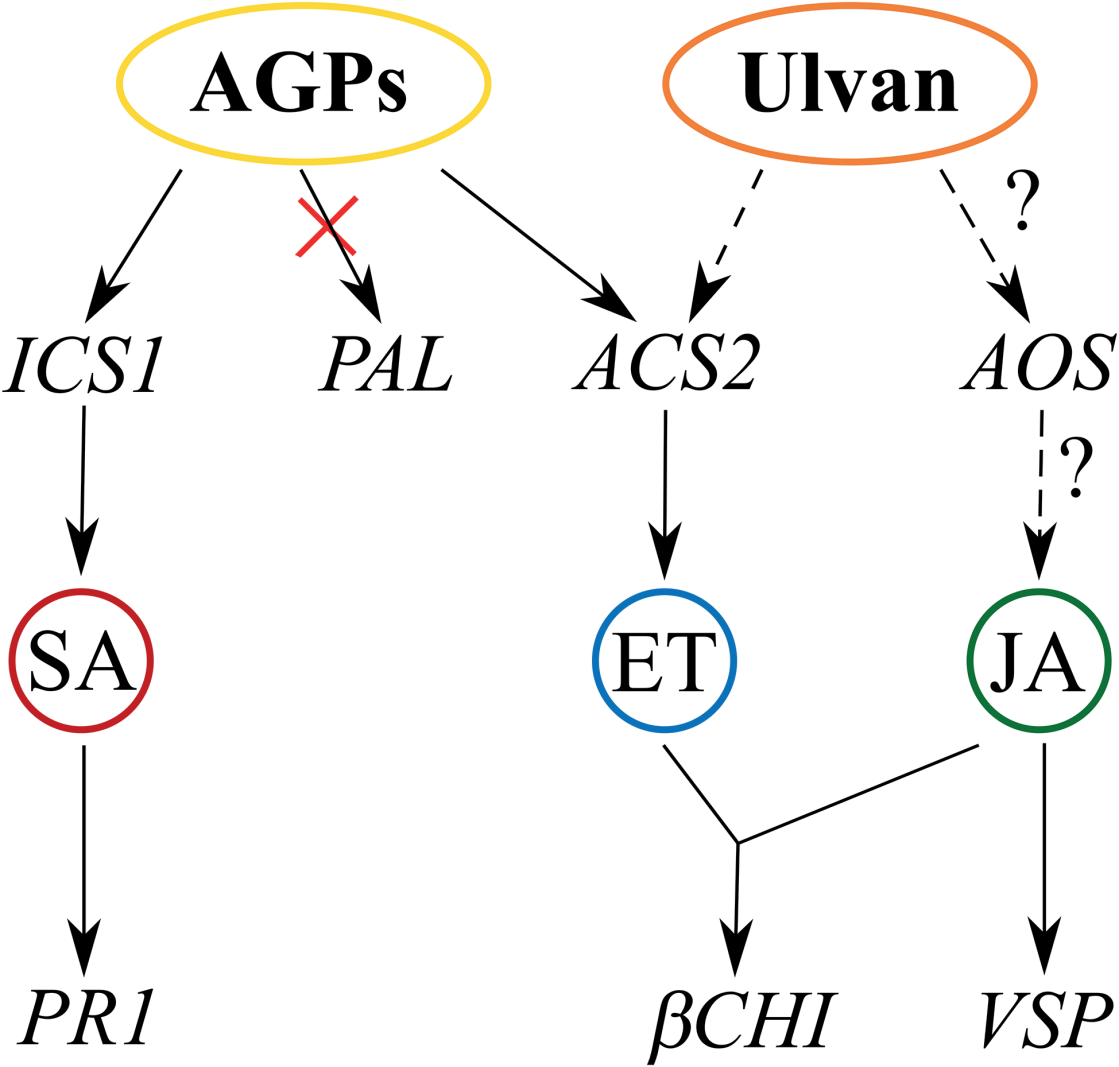
Proposed mechanism of AGP-like enriched fraction (AGPs) and ulvan action based on transcription of signaling pathway marker genes. AGPs activate the salicylic acid signaling pathway *via isochorismate synthase 1* and the ethylene signaling pathway *via ACC synthase*. Ulvan activates the ethylene signaling pathway based on gene expression of *βCHI* and probably also jasmonic acid signaling pathway based on *VSP* expression. Full arrows indicate proven involvement of the pathways, and dashed arrows indicate likely involvement of the pathways.

In conclusion, our study makes an important contribution to the understanding of the mechanisms behind the elicitor activity of *U. lactuca* extracts recently introduced in agriculture. In addition to the well-described polysaccharide ulvan, *U. lactuca* contains other compounds that elicit even stronger defenses against pathogens. We were able to prepare an AGP-like enriched fraction that efficiently induced resistance to the hemibiotrophic fungal pathogen *L. maculans* in cotyledons of *B. napus*. Examination of the signaling events revealed that the triggered defense mechanisms were regulated by H_2_O_2_, SA, and ET signaling. Proposed mechanisms of actions for both AGPs and ulvan are presented in the Figure 6. Since AGPs showed higher efficiency than ulvan, AGPs may have the potential to become a component of plant protection products in the future. Moreover, for their possible future application, our following research will be focused on increased penetration of AGPs to the plants, testing oligosaccharides produced from AGPs, and assessing their effect also on other pathosystems including monocot plants, which differ in their defense signaling.

## Data Availability Statement

The original contributions presented in the study are included in the article/**Supplementary Material**; further inquiries can be directed to the corresponding author.

## Author Contributions

TP contributed to data collection, designing research, and analyzing the data and writing the manuscript. BJ contributed to data collection, designing plant experiments, analyzing the data, and editing the manuscript. SH contributed to data collection (saccharide analysis). J-CY and VF contributed to designing research. LB contributed to designing research and editing the manuscript. PL contributed to data collection, designing research, and editing the manuscript. All authors contributed to the article and approved the submitted version.

## Funding

The work was supported from European Regional Development Fund-Project “Centre for Experimental Plant Biology” (no. CZ.02.1.01/0.0/0.0/16_019/0000738) and by the grant of Specific University Research (A1_FPBT_2020_001).

## Conflict of Interest

The authors declare that the research was conducted in the absence of any commercial or financial relationships that could be construed as a potential conflict of interest.

## Publisher’s Note

All claims expressed in this article are solely those of the authors and do not necessarily represent those of their affiliated organizations, or those of the publisher, the editors and the reviewers. Any product that may be evaluated in this article, or claim that may be made by its manufacturer, is not guaranteed or endorsed by the publisher.

## References

[ref1] Abouraïcha E. El Alaoui-Talibi Z. El Boutachfaiti R. Petit E. Courtois B. Courtois J.. (2015). Induction of natural defense and protection against *Penicillium expansum* and *Botrytis cinerea* in apple fruit in response to bioelicitors isolated from green algae. Sci. Hortic. 181, 121–128. doi: 10.1016/j.scienta.2014.11.002

[ref2] Ali O. Ramsubhag A. Jayaraman J. (2021). Biostimulant properties of seaweed extracts in plants, implications towards sustainable crop production. Plan. Theory 10:531. doi: 10.3390/plants10030531, PMID: 33808954 PMC8000310

[ref3] Allègre M. Héloir M.-C. Trouvelot S. Daire X. Pugin A. Wendehenne D.. (2009). Are grapevine stomata involved in the elicitor-induced protection against downy mildew? MPMI 22, 977–986. doi: 10.1094/MPMI-22-8-0977, PMID: 19589073

[ref4] Aquino R. S. Grativol C. Mourão P. A. S. (2011). Rising from the sea, correlations between sulfated polysaccharides and salinity in plants. PLoS One 6:e18862. doi: 10.1371/journal.pone.0018862, PMID: 21552557 PMC3084243

[ref5] Aziz A. Poinssot B. Daire X. Adrian M. Bézier A. Lambert B.. (2003). Laminarin elicits defense responses in grapevine and induces protection against *Botrytis cinerea* and *Plasmospara viticola*. MPMI 16, 1118–1128. doi: 10.1094/MPMI.2003.16.12.1118, PMID: 14651345

[ref6] Balesdent M. H. Attard A. Ansan-Melayah D. Delourme R. Renard M. Rouxel T. (2001). Genetic control and host range of avirulence toward *Brassica napus* cultivars Quinta and jet Neuf in *Leptosphaeria maculans*. Phytopathology 91, 70–76. doi: 10.1094/PHYTO.2001.91.1.70, PMID: 18944280

[ref7] Barna B. Fodor J. Harrach B. D. Pogány M. Király Z. (2012). The Janus face of reactive oxygen species in resistance and susceptibility of plants to necrotrophic and biotrophic pathogens. Plant Physiol. Biochem. 59, 37–43. doi: 10.1016/j.plaphy.2012.01.014, PMID: 22321616

[ref8] Bartels D. Baumann A. Maeder M. Geske T. Heise E. M. von Schwartzenberg K.. (2017). Evolution of plant cell wall: arabinogalactan-proteins from three moss genera show structural differences compared to seed plants. Carbohydr. Polym. 163, 227–235. doi: 10.1016/j.carbpol.2017.01.043, PMID: 28267501

[ref9] Bartels D. Classen B. (2017). Structural investigations on arabinogalactan-proteins from a lycophyte and different monilophytes (ferns) in the evolutionary context. Carbohydr. Polym. 172, 342–351. doi: 10.1016/j.carbpol.2017.05.031, PMID: 28606543

[ref10] Battacharyya D. Babgohari M. Z. Rathor P. Prithiviraj B. (2015). Seaweed extracts as biostimulants in horticulture. Sci. Hortic. 196, 39–48. doi: 10.1016/j.scienta.2015.09.012, PMID: 34866802

[ref11] Becker M. G. Zhang X. Walker P. L. Wan J. C. Millar J. L. Khan D.. (2017). Transcriptome analysis of the *Brassica napus–Leptosphaeria maculans* pathosystem identifies receptor, signaling and structural genes underlying plant resistance. Plant J. 90, 573–586. doi: 10.1111/tpj.13514, PMID: 28222234

[ref12] Benedetti M. Pontiggia D. Raggia S. Cheng Z. Scalonia F. Ferrari S.. (2015). Plant immunity triggered by engineered *in vivo* releaseof oligogalacturonides, damage-associated molecular patterns. PNAS 112, 5533–5538. doi: 10.1073/pnas.1504154112, PMID: 25870275 PMC4418913

[ref13] Bigeard J. Colcombet J. Hirt H. (2015). Signaling mechanisms in pattern-triggered immunity (PTI). Mol. Plant 8, 521–539. doi: 10.1016/j.molp.2014.12.022, PMID: 25744358

[ref14] Blumenkrantz N. Asboe-Hansen G. (1973). New method for quantitative determination of uronic acids. Anal. Biochem. 54, 484–489. doi: 10.1016/0003-2697(73)90377-1, PMID: 4269305

[ref15] Boller T. Felix G. (2009). A renaissance of elicitors, perception of microbe-associated molecular patterns and danger signals by pattern-recognition receptors. Annu. Rev. Plant Biol. 60, 379–406. doi: 10.1146/annurev.arplant.57.032905.105346, PMID: 19400727

[ref16] Bollig K. Lamshöft M. Schweimer K. Marner F.-J. Budzikiewicz H. Waffenschmidt S. (2007). Structural analysis of linear hydroxyproline-bound *O*-glycans of *Chlamydomonas reinhardtii*—conservation of the inner core in *Chlamydomonas* and land plants. Carbohydr. Res. 342, 2557–2566. doi: 10.1016/j.carres.2007.08.008, PMID: 17854785

[ref701] Borba M. C. Stadnik M. B. Stadnik M. J. (2019). Ulvan enhances seedling emergence and reduces Fusarium wilt severity in common bean (*Phaseolus vulgaris* L.). Crop Prot. 118, 66–71. doi: 10.1016/j.cropro.2018.12.014

[ref17] Borba M. C. Velho A. C. Maia-Grondard A. Baltenweck R. Magnin-Robert M. Randoux B.. (2021). The algal polysaccharide Ulvan induces resistance in wheat against *Zymoseptoria tritici* without major alteration of leaf Metabolome. Front. Plant Sci. 12:703712. doi: 10.3389/fpls.2021.703712, PMID: 34552606 PMC8450535

[ref18] Bürger M. Chory J. (2019). Stressed out about hormones, how plants orchestrate immunity. Cell Host Microbe 26, 163–172. doi: 10.1016/j.chom.2019.07.006, PMID: 31415749 PMC7228804

[ref19] Burketova L. Trda L. Ott P. G. Valentova O. (2015). Bio-based resistance inducers for sustainable plant protection against pathogens. Biotechnol. Adv. 33, 994–1004. doi: 10.1016/j.biotechadv.2015.01.004, PMID: 25617476

[ref20] Capek P. Matulová M. Combourieu B. (2008). The extracellular proteoglycan produced by *Rhodella grisea*. Int. J. Biol. Macromol. 43, 390–393. doi: 10.1016/j.ijbiomac.2008.07.015, PMID: 18706924

[ref21] Carvalho F. P. (2006). Agriculture, pesticides, food security and food safety. Environ. Sci. Pol. 9, 685–692. doi: 10.1016/j.envsci.2006.08.002, PMID: 35395011

[ref22] Castellanos-Barriga L. G. Santacruz-Ruvalcaba F. Hernández-Carmona G. Ramírez-Briones E. Hernández-Herrera R. M. (2017). Effect of seaweed liquid extracts from *Ulva lactuca* on seedling growth of mung bean (*Vigna radiata*). J. Appl. Phycol. 29, 2479–2488. doi: 10.1007/s10811-017-1082-x

[ref23] Chapman J. M. Muhlemann J. K. Gayomba S. R. Muday G. K. (2019). RBOH-dependent ROS synthesis and ROS scavenging by plant specialized metabolites to modulate plant development and stress responses. Chem. Res. Toxicol. 32, 370–396. doi: 10.1021/acs.chemrestox.9b00028, PMID: 30781949 PMC6857786

[ref24] Chbani A. Majed S. Mawlawi H. (2015). Mineral content of mediterranean seaweeds, *Padina pavonica* L. (Pheophytae), *Ulva lactuca* L. and *Ulva linza* L. (Chlorophytae) for biofertilizing use. Int. J. Hortic. Sci. Technol. 2, 133–140. doi: 10.22059/IJHST.2015.56430

[ref25] Chen Q. Chen Z. Lu L. Jin H. Sun L. Yu Q.. (2013). Interaction between abscisic acid and nitric oxide in PB90-induced catharanthine biosynthesis of *catharanthus roseus* cell suspension cultures. Biotechnol. Prog. 29, 994–1001. doi: 10.1002/btpr.1738, PMID: 23554409

[ref26] Cluzet S. Torregrosa C. Jacquet C. Lafitte C. Fournier J. Mercier L.. (2004). Gene expression profiling and protection of *Medicago truncatula* against a fungal infection in response to an elicitor from green algae *Ulva* spp. Plant Cell Environ. 27, 917–928. doi: 10.1111/j.1365-3040.2004.01197.x

[ref27] Cook J. Zhang J. Norrie J. Blal B. Cheng Z. (2018). Seaweed extract (Stella Maris®) activates innate immune responses in *Arabidopsis thaliana* and protects host against bacterial pathogens. Mar. Drugs 16:221. doi: 10.3390/md16070221, PMID: 29958402 PMC6071235

[ref28] Cramer G. R. Läuchli A. Polito V. S. (1985). Displacement of Ca^2+^ by Na^+^ from the plasmalemma of root cells. Plant Physiol. 79, 207–211. doi: 10.1104/pp.79.1.207, PMID: 16664372 PMC1074853

[ref29] Divya K. Roja N. Padal S. B. (2015). Influence of seaweed liquid fertilizer of *Ulva lactuca* on the seed germination, growth, productivity of *Abelmoschus esculentus* (L.). Int. J. Pharmacol. Res. 5, 344–356.

[ref30] Dominguez H. Loret E. P. (2019). *Ulva lactuca*, a source of troubles and potential riches. Mar. Drugs 17:357. doi: 10.3390/md17060357, PMID: 31207947 PMC6627311

[ref31] Domozych D. Ciancia M. Fangel J. U. Mikkelsen M. D. Ulvskov P. Willats W. G. T. (2012). The cell walls of green algae, a journey through evolution and diversity. Front. Plant Sci. 3:82. doi: 10.3389/fpls.2012.00082, PMID: 22639667 PMC3355577

[ref32] Domozych D. S. Elliott L. Kiemle S. N. Gretz M. R. (2007). *Pleurotaenium trabecula*, a desmid of wetland biofilms: the extracellular matrix and adhesion mechanisms. J. Phycol. 43, 1022–1038. doi: 10.1111/j.1529-8817.2007.00389.x

[ref33] Domozych D. S. Sørensen I. Pettolino F. A. Bacic A. Willats W. G. T. (2010). The cell wall polymers of the charophycean green alga *Chara corallina*: immunobinding and biochemical screening. Int. J. Plant Sci. 171, 345–361. doi: 10.1086/651227

[ref34] Domozych D. S. Wilson R. Domozych C. R. (2009). Photosynthetic eukaryotes of freshwater wetland biofilms: adaptations and structural characteristics of the extracellular matrix in the green alga, *Cosmarium reniforme* (Zygnematophyceae, Streptophyta). J. Eukaryot. Microbiol. 56, 314–322. doi: 10.1111/j.1550-7408.2009.00392.x, PMID: 19602077

[ref35] du Jardin P. Xu L. Geelen D. (2020). “Agricultural functions and action mechanisms of plant biostimulants (PBs)” in The Chemical Biology of Plant Biostimulants. eds. Geelen D. Xu L. (Chichester, UK: John Wiley, and Sons, Ltd.), 1–30.

[ref36] Eder M. Tenhaken R. Driouich A. Lütz-Meindl U. (2008). Occurrence and characterization of arabinogalactan-like proteins and hemicelluloses in *Micrasterias* (Streptophyta). J. Phycol. 44, 1221–1234. doi: 10.1111/j.1529-8817.2008.00576.x, PMID: 27041719

[ref37] El Boukhari M. E. M. Barakate M. Bouhia Y. Lyamlouli K. (2020). Trends in seaweed extract based biostimulants, manufacturing process and beneficial effect on soil-plant systems. Plan. Theory 9:359. doi: 10.3390/plants9030359, PMID: 32178418 PMC7154814

[ref38] Estevez J. M. Fernández P. V. Kasulin L. Dupree P. Ciancia M. (2009). Chemical and in situ characterization of macromolecular components of the cell walls from the green seaweed *Codium fragile*. Glycobiology 19, 212–228. doi: 10.1093/glycob/cwn101, PMID: 18832454

[ref39] Estevez J. M. Leonardi P. I. Alberghina J. S. (2008). Cell wall carbohydrate epitopes in the green alga *Oedogonium Bharuchae* F*. Minor* (Oedogoniales, Chlorophyta)1. J. Phycol. 44, 1257–1268. doi: 10.1111/j.1529-8817.2008.00568.x, PMID: 27041722

[ref40] Fernández P. V. Ciancia M. Miravalles A. B. Estevez J. M. (2010). Cell-wall polymer mapping in the coenocytic macroalga *Codium Vermilara* (Bryopsidales, Chlorophyta). J. Phycol. 46, 456–465. doi: 10.1111/j.1529-8817.2010.00821.x

[ref41] Fernández P. V. Raffo M. P. Alberghina J. Ciancia M. (2015). Polysaccharides from the green seaweed *Codium decorticatum*. Structure and cell wall distribution. Carbohydr. Polym. 117, 836–844. doi: 10.1016/j.carbpol.2014.10.039, PMID: 25498707

[ref42] Freitas M. B. Stadnik M. J. (2012). Race-specific and ulvan-induced defense responses in bean (*Phaseolus vulgaris*) against *Colletotrichum lindemuthianum*. Physiol. Mol. Plant Pathol. 78, 8–13. doi: 10.1016/j.pmpp.2011.12.004

[ref43] Freitas M. B. Stadnik M. J. (2015). Ulvan-induced resistance in *Arabidopsis thaliana* against *Alternaria brassicicola* requires reactive oxygen species derived from NADPH oxidase. Physiol. Mol. Plant Pathol. 90, 49–56. doi: 10.1016/j.pmpp.2015.03.002

[ref44] Gireesh R. Haridevi C. K. Salikutty J. (2011). Effect of *Ulva lactuca* extract on growth and proximate composition of *Vigna unguiculata* (L.) Walp. J. Res. Biol. 8, 624–630.

[ref45] Hahn M. G. Darvill A. G. Albersheim P. (1981). Host-pathogen interactions, XIX. The endogenous elicitor, a fragment of a plant cell wall polysaccharide that elicits phytoalexin accumulation in soybeans. Plant Physiol. 68, 1161–1169. doi: 10.1104/pp.68.5.1161, PMID: 16662068 PMC426062

[ref46] Hahn T. Schulz M. Stadtmüller R. Zayed A. Muffler K. Lang S.. (2016). Cationic dye for the specific determination of sulfated polysaccharides. Anal. Lett. 49, 1948–1962. doi: 10.1080/00032719.2015.1126839, PMID: 29105785

[ref47] Happ K. Classen B. (2019). Arabinogalactan-proteins from the liverwort *Marchantia polymorpha* L., a member of a basal land plant lineage, are structurally different to those of angiosperms. Plan. Theory 8:460. doi: 10.3390/plants8110460, PMID: 31671872 PMC6918356

[ref48] Hardy M. R. Townsend R. R. Leeab Y. C. (1988). Monosaccharide analysis of glycoconjugates by anion exchange chromatography with pulsed amperometric detection. Anal. Biochem. 170, 54–62. doi: 10.1016/0003-2697(88)90089-9, PMID: 3389518

[ref702] Hernández-Herrera R. M. Santacruz-Ruvalcaba F. Ruiz-López M. A. Norrie J. Hernández-Carmona G. (2014). Effect of liquid seaweed extracts on growth of tomato seedlings (*Solanum lycopersicum* L.). J. Appl. Phycol. 26, 619–628. doi: 10.1007/s10811-013-0078-4, PMID: 18832454

[ref49] Hernández-Herrera R. M. Santacruz-Ruvalcaba F. Zañudo-Hernández J. Hernández-Carmona G. (2016). Activity of seaweed extracts and polysaccharide-enriched extracts from *Ulva lactuca* and *Padina gymnospora* as growth promoters of tomato and mung bean plants. J. Appl. Phycol. 28, 2549–2560. doi: 10.1007/s10811-015-0781-4

[ref50] Hervé C. Siméon A. Jam M. Cassin A. Johnson K. L. Salmeán A. A.. (2015). Arabinogalactan proteins have deep roots in eukaryotes, identification of genes and epitopes in brown algae and their role in *Fucus serratus* embryo development. New Phytol. 209, 1428–1441. doi: 10.1111/nph.13786 26667994

[ref51] Huang H. Ullah F. Zhou D.-X. Yi M. Zhao Y. (2019). Mechanisms of ROS regulation of plant development and stress responses. Front. Plant Sci. 10:800. doi: 10.3389/fpls.2019.00800, PMID: 31293607 PMC6603150

[ref52] Jamiolkowska A. (2020). Natural compounds as elicitors of plant resistance against diseases and new biocontrol strategies. Agronomy 10:173. doi: 10.3390/agronomy100(2017)3

[ref53] Jasso-Robles F. I. Gonzalez M. E. Pieckenstain F. L. Ramírez-García J. M. Guerrero-González M. Jiménez-Bremont J. F.. (2020). Decrease of *Arabidopsis* PAO activity entails increased RBOH activity, ROS content and altered responses to *pseudomonas*. Plant Sci. 292:110372. doi: 10.1016/j.plantsci.2019.110372, PMID: 32005378

[ref54] Jaulneau V. Lafitte C. Corio-Costet M.-F. Stadnik M. J. Salamagne S. Briand X.. (2011). An *Ulva armoricana* extract protects plants against three powdery mildew pathogens. Eur. J. Plant Pathol. 131, 393–401. doi: 10.1007/s10658-011-9816-0

[ref55] Jaulneau V. Lafitte C. Jacquet C. Fournier S. Salamagne S. Briand X.. (2010). Ulvan, a sulfated polysaccharide from green algae, activates plant immunity through the jasmonic acid signaling pathway. J. Biomed. Biotechnol. 2010:525291. doi: 10.1155/2010/525291, PMID: 20445752 PMC2860583

[ref56] Jindřichová B. Burketová L. Novotná Z. (2014). Novel properties of antimicrobial peptide anoplin. Biochem. Biophys. Res. Commun. 444, 520–524. doi: 10.1016/j.bbrc.2014.01.097, PMID: 24472551

[ref57] Jindřichová B. Fodor J. Šindelářová M. Burketová L. Valentová O. (2011). Role of hydrogen peroxide and antioxidant enzymes in the interaction between a hemibiotrophic fungal pathogen, *Leptosphaeria maculans*, and oilseed rape. Environ. Exp. Bot. 72, 149–156. doi: 10.1016/j.envexpbot.2011.02.018

[ref58] Jones J. D. G. Dangl J. L. (2006). The plant immune system. Nature 444, 323–329. doi: 10.1038/nature05286, PMID: 17108957

[ref59] Kámán-Tóth E. Dankó T. Gullner G. Bozsó Z. Palkovics L. Pogány M. (2019). Contribution of cell wall peroxidase- and NADPH oxidase-derived reactive oxygen species to *Alternaria brassicicola*-induced oxidative burst in *Arabidopsis*. Mol. Plant Pathol. 20, 485–499. doi: 10.1111/mpp.12769, PMID: 30426643 PMC6637864

[ref60] Kidgell J. T. Magnusson M. de Nys R. Glasson C. R. K. (2019). Ulvan, a systematic review of extraction, composition and function. Algal Res. 39:101422. doi: 10.1016/j.algal.2019.101422

[ref61] Kim P. D. Šašek V. Burketová L. Čopíková J. Synytsya A. Jindřichová B.. (2013). Cell Wall components of *Leptosphaeria maculans* enhance resistance of *Brassica napus*. J. Agric. Food Chem. 61, 5207–5214. doi: 10.1021/jf401221v, PMID: 23638999

[ref62] Klarzynski O. Plesse B. Joubert J.-M. Yvin J.-C. Kopp M. Kloareg B.. (2000). Linear b-1,3 Glucans are elicitors of defense responses in tobacco. Plant Physiol. 124, 1027–1038. doi: 10.1104/pp.124.3.1027, PMID: 11080280 PMC59202

[ref63] Lahaye P. A. Epstein E. (1969). Salt toleration by plants, enhancement with calcium. Science 166, 395–396. doi: 10.1126/science.166.3903.395, PMID: 17796555

[ref64] Lahaye P. A. Epstein E. (1971). Calcium and salt toleration by bean plants. Physiol. Plant. 25, 213–218. doi: 10.1111/j.1399-3054.1971.tb01430.x, PMID: 17796555

[ref703] Lahaye P. A. Robic E. (2007). Structure and functional properties of ulvan, a polysaccharide from green seaweeds. Biomacromolecules 6, 1765–1774. doi: 10.1021/bm061185q, PMID: 17458931

[ref65] Lamport D. T. A. Tan L. Held M. Kieliszewski M. J. (2018). The role of the primary cell wall in plant morphogenesis. Int. J. Mol. Sci. 19:2674. doi: 10.3390/ijms19092674, PMID: 30205598 PMC6165521

[ref66] Lamport D. T. A. Várnai P. (2013). Periplasmic arabinogalactan glycoproteins act as a calcium capacitor that regulates plant growth and development. New Phytol. 197, 58–64. doi: 10.1111/nph.12005, PMID: 23106282

[ref67] Lamport D. T. A. Varnai P. Seal C. E. (2014). Back to the future with the AGP–Ca^2+^ flux capacitor. Ann. Bot. 114, 1069–1085. doi: 10.1093/aob/mcu161, PMID: 25139429 PMC4195563

[ref68] Lee K. J. D. Sakata Y. Mau S. L. Pettolino F. Bacic A. Quatrano R. S.. (2005). Arabinogalactan proteins are required for apical cell extension in the moss *Physcomitrella patens*. Plant Cell 17, 3051–3065. doi: 10.1105/tpc.105.034413, PMID: 16199618 PMC1276029

[ref69] Levy-Ontman O. Arad S. Harvey D. J. Parsons T. B. Fairbanks A. Tekoah Y. (2011). Unique *N*-glycan moieties of the 66-kDa cell wall glycoprotein from the red microalga *Porphyridium* sp. J. Biol. Chem. 286, 21340–21352. doi: 10.1074/jbc.M110.175042, PMID: 21515680 PMC3122194

[ref70] Lin Z. Lin Z. Li H. Shen S. (2012). Sequences analysis of ITS region and 18S rDNA of *Ulva*. ISRN Bot. 2012:468193. doi: 10.5402/2012/468193

[ref71] Lipková N. Medo J. Artimová R. Maková J. Petrová J. Javoreková S.. (2021). Growth promotion of rapeseed (*Brassica napus* L.) and blackleg disease (*Leptosphaeria maculans*) suppression mediated by Endophytic bacteria. Agronomy 11:1966. doi: 10.3390/agronomy11101966

[ref72] Lopez-Hernandez F. Tryfona T. Rizza A. Yu X. L. Harris M. O. B. Webb A. A. R.. (2020). Calcium binding by Arabinogalactan polysaccharides is important for normal plant development. Plant Cell 32, 3346–3369. doi: 10.1105/tpc.20.00027, PMID: 32769130 PMC7534474

[ref73] Ma Y. Yan C. Li H. Wu W. Liu Y. Wang Y.. (2017). Bioinformatics prediction and evolution analysis of arabinogalactan proteins in the plant kingdom. Front. Plant Sci. 8:66. doi: 10.3389/fpls.2017.00066, PMID: 28184232 PMC5266747

[ref74] Ma Y. Zeng W. Bacic A. Johnson K. (2018). “AGPs through time and space” in Annual plant reviews online. ed. Roberts J. A. (Chichester, UK: John Wiley & Sons, Ltd.), 767–804.

[ref75] Mareri L. Romi M. Cai G. (2019). Arabinogalactan proteins, actors or spectators during abiotic and biotic stress in plants? Plant. Biosystems 153, 173–185. doi: 10.1080/11263504.2018.1473525

[ref76] Martin R. L. Le Boulch P. Clin P. Schwarzenberg A. Yvin J.-C. Andrivon D.. (2020). A comparison of PTI defense profiles induced in *Solanum tuberosum* by PAMP and non-PAMP elicitors shows distinct, elicitor-specific responses. PLoS One 15:e0236633. doi: 10.1371/journal.pone.0236633, PMID: 32785249 PMC7423108

[ref77] Mathieu-Rivet E. Kiefer-Meyer M.-C. Vanier G. Ovide C. Burel C. Lerouge P.. (2014). Protein *N*-glycosylation in eukaryotic microalgae and its impact on the production of nuclear expressed biopharmaceuticals. Front. Plant Sci. 5:359. doi: 10.3389/fpls.2014.00359, PMID: 25183966 PMC4135232

[ref78] Mercier L. Lafitte C. Borderies G. Briand X. Marie-Thérèse Esquerré-Tugayé M.-T. Fournier J. (2001). The algal polysaccharide carrageenans can act as an elicitor of plant defence. New Phytol. 149, 43–51. doi: 10.1046/j.1469-8137.2001.00011.x, PMID: 33853239

[ref79] Mizukami A. G. Inatsugi R. Jiao J. Kotake T. Kuwata K. Ootani K.. (2016). The AMOR arabinogalactan sugar chain induces pollen-tube competency to respond to ovular guidance. Curr. Biol. 26, 1091–1097. doi: 10.1016/j.cub.2016.02.040, PMID: 27068416

[ref80] Mócsai R. Figl R. Troschl C. Strasser R. Svehla E. Windwarder M.. (2019). *N*-glycans of the microalga *Chlorella vulgaris* are of the oligomannosidic type but highly methylated. Sci. Rep. 9:331. doi: 10.1038/s41598-018-36884-1, PMID: 30674946 PMC6344472

[ref81] Morales J. Kadota Y. Zipfel C. Molina A. Torres M.-A. (2016). The *Arabidopsis* NADPH oxidases RbohD and RbohF display differential expression patterns and contributions during plant immunity. J. Exp. Bot. 67, 1663–1676. doi: 10.1093/jxb/erv558, PMID: 26798024

[ref82] Nabti E. Jha B. Hartmann A. (2017). Impact of seaweeds on agricultural crop production as biofertilizer. Int. J. Environ. Sci. Technol. 14, 1119–1134. doi: 10.1007/s13762-016-1202-1

[ref83] Nagel A. Sirisakulwat S. Carle R. Neidhart S. (2014). An acetate−hydroxide gradient for the quantitation of the neutral sugar and Uronic acid profile of Pectins by HPAEC-PAD without Postcolumn pH adjustment. J. Agric. Food Chem. 62, 2037–2048. doi: 10.1021/jf404626d, PMID: 24547908

[ref84] Neik T. X. Ama J. Barbetti M. Edwards D. Batley J. (2020). Understanding host–pathogen interactions in *Brassica napus* in the Omics era. Plan. Theory 9:1336. doi: 10.3390/plants9101336, PMID: 33050509 PMC7599536

[ref85] Neik T. X. Barbetti M. Batley J. (2017). Current status and challenges in identifying disease resistance genes in *Brassica napus*. Front. Plant Sci. 8:1788. doi: 10.3389/fpls.2017.01788, PMID: 29163558 PMC5681527

[ref86] Nguema-Ona E. Coimbra S. Vicré-Gibouin M. Mollet J.-C. Driouich A. (2012). Arabinogalactan-proteins in root and pollen tube cells, distribution and functional properties. Ann. Bot. 110, 383–404. doi: 10.1093/aob/mcs143, PMID: 22786747 PMC3394660

[ref87] Nguema-Ona E. Vicré-Gibouin M. Cannesan M.-A. Driouich A. (2013). Arabinogalactan proteins in root–microbe interactions. Trends Plant Sci. 18, 440–449. doi: 10.1016/j.tplants.2013.03.006, PMID: 23623239

[ref88] Nováková M. Kim P. D. Šašek V. Burketová L. Jindřichová B. Šantrůček J.. (2016). Separation and identification of candidate protein elicitors from the cultivation medium of *Leptosphaeria maculans* inducing resistance in *Brassica napus*. Biotechnol. Prog. 32, 918–928. doi: 10.1002/btpr.2266, PMID: 27009514

[ref89] Nováková M. Šašek V. Dobrev P. I. Valentová O. Burketová L. (2014). Plant hormones in defense response of *Brassica napus* to *Sclerotinia sclerotiorum*—reassessing the role of salicylic acid in the interaction with a necrotroph. Plant Physiol. Biochem. 80, 308–317. doi: 10.1016/j.plaphy.2014.04.019, PMID: 24837830

[ref90] Ogawa K. Arai M. Naganawa H. Ikeda Y. Kondo S. (2001). A new β-d-Galactan having 3-*O*-methyl-d-galactose from *Chlorella vulgaris*. J. Appl. Glycosci. 48, 325–330. doi: 10.5458/jag.48.325

[ref91] Palacio-López K. Tinaz B. Holzinger A. Domozych D. S. (2019). Arabinogalactan proteins and the extracellular matrix of charophytes: a sticky business. Front. Plant Sci. 10:447. doi: 10.3389/fpls.2019.00447, PMID: 31031785 PMC6474363

[ref92] Paulert R. Ascrizzi R. Malatesta S. Berni P. Noseda M. D. Mazetto de Carvalho M.. (2021). *Ulva intestinalis* extract acts as biostimulant and modulates metabolites and hormone balance in basil (*Ocimum basilicum* L.) and parsley (*Petroselinum crispum* L.). Plan. Theory 10:1391. doi: 10.3390/plants10071391, PMID: 34371594 PMC8309453

[ref93] Paulert R. Ebbinghaus D. Urlass C. Moerschbacher B. M. (2010). Priming of the oxidative burst in rice and wheat cell cultures by ulvan, a polysaccharide from green macroalgae, and enhanced resistance against powdery mildew in wheat and barley plants. Plant Pathol. 59, 634–642. doi: 10.1111/j.1365-3059.2010.02300.x

[ref94] Paulert R. Talamini V. Cassolato J. E. F. Duarte M. E. R. Noseda M. D. Smania A.. (2009). Effects of sulfated polysaccharide and alcoholic extracts from green seaweed *Ulva fasciata* on anthracnose severity and growth of common bean (*Phaseolus vulgaris* L). J. Plant Dis. Prot. 116, 263–270. doi: 10.1007/BF03356321

[ref704] Pfeifer L. Classen B. (2020). The cell wall of seagrasses: Fascinating, peculiar and a blank canvas for future research. Front. Plant Sci. 11:588754. doi: 10.3389/fpls.2020.588754 33193541 PMC7644952

[ref95] Pfeifer L. Shafee T. Johnson K. L. Bacic A. Classen B. (2020). Arabinogalactan-proteins of *Zostera marina* L. contain unique glycan structures and provide insight into adaption processes to saline environments. Sci. Rep. 10:8232. doi: 10.1038/s41598-020-65135-5, PMID: 32427862 PMC7237498

[ref96] Pfeifer L. Utermöhlen J. Happ K. Permann C. Holzinger A. Schwartzenberg K.. (2021). Search for evolutionary roots of land plant arabinogalactan-proteins in charophytes: presence of a rhamnogalactan-protein in *Spirogyra pratensis* (Zygnematophyceae). Plant J. 109, 568–584. doi: 10.1111/tpj.15577, PMID: 34767672 PMC7612518

[ref97] Přerovská T. Henka S. Bleha R. Spiwok V. Gillarová S. Yvin J.-C.. (2021). Arabinogalactan-like glycoproteins from *Ulva lactuca* (Chlorophyta) show unique features compared to land plants AGPs. J. Phycol. 57, 619–635. doi: 10.1111/jpy.13121, PMID: 33338254

[ref98] Raboanatahiry N. Li H. Yu L. Li M. (2021). Rapeseed (*Brassica napus*), processing, utilization, and genetic improvement. Agronomy 11:1776. doi: 10.3390/agronomy11091776, PMID: 30385414

[ref99] Ramkissoon A. Ramsubhag A. Jayaraman J. (2017). Phytoelicitor activity of three Caribbean seaweed species on suppression of pathogenic infections in tomato plants. J. Appl. Phycol. 29, 3235–3244. doi: 10.1007/s10811-017-1160-0

[ref100] Ray D. K. Mueller N. D. West P. C. Foley J. A. (2013). Yield trends are insufficient to double global crop production by 2050. PLoS One 8:e66428. doi: 10.1371/journal.pone.0066428, PMID: 23840465 PMC3686737

[ref101] Renzaglia K. S. Villareal J. C. Piatkowski B. T. Lucas J. R. Merced A. (2017). Hornwort stomata, architecture, and fate of shared with 400 million year old fossil plants without leaves. Plant Physiol. 174, 788–797. doi: 10.1104/pp.17.00156, PMID: 28584065 PMC5462037

[ref102] Robic A. Bertrand D. Sassi J.-F. Lerat Y. Lahaye M. (2009). Determination of the chemical composition of ulvan, a cell wall polysaccharide from *Ulva* spp. (Ulvales, Chlorophyta) by FT-IR and chemometrics. J. Appl. Phycol. 21, 451–456. doi: 10.1007/s10811-008-9390-9

[ref103] Rouphael Y. Colla G. (2020). Editorial, biostimulants in agriculture. Front. Plant Sci. 11:40. doi: 10.3389/fpls.2020.00040, PMID: 32117379 PMC7010726

[ref104] Šašek V. Nováková M. Dobrev P. I. Valentová O. Burketová L. (2012a). Beta-aminobutyric acid protects *Brassica napus* plants from infection by *Leptosphaeria maculans*. Resistance induction or a direct antifungal effect? Eur. J. Plant Pathol. 133, 279–289. doi: 10.1007/s10658-011-9897-9

[ref105] Šašek V. Nováková M. Jindřichová B. Bóka K. Valentová O. Burketová L. (2012b). Recognition of avirulence gene AvrLm1 from hemibiotrophic ascomycete *Leptosphaeria maculans* triggers salicylic acid and ethylene signaling in *Brassica napus*. Mol. Plant-Microbe Interact. 25, 1238–1250. doi: 10.1094/MPMI-02-12-0033-R, PMID: 22624662

[ref106] Seifert G. J. Roberts K. (2007). The biology of arabinogalactan proteins. Annu. Rev. Plant Biol. 58, 137–161. doi: 10.1146/annurev.arplant.58.032806.103801, PMID: 17201686

[ref107] Sharma H. S. S. Fleming C. Selby C. Rao J. R. Martin T. (2014). Plant biostimulants, a review on the processing of macroalgae and use of extracts for crop management to reduce abiotic and biotic stresses. J. Appl. Phycol. 26, 465–490. doi: 10.1007/s10811-013-0101-9

[ref108] Shefer S. Lebendiker M. Finkelshtein A. Chamovitz D. A. Golberg A. (2022). Ulvan crude extract’s chemical and biophysical profile and its effect as a biostimulant on *Arabidopsis thaliana*. Algal Res. 62:102609. doi: 10.1016/j.algal.2021.102609

[ref109] Shoubaky G. Salem E. A. (2016). Effect of abiotic stress on endogenous phytohormones profile in some seaweeds. Int. J. Pharmacogn. Phytochem. Res. 8, 124–134.

[ref110] Showalter A. M. (2001). Arabinogalactan-proteins, structure, expression and function. Cell. Mol. Life Sci. 58, 1399–1417. doi: 10.1007/PL00000784, PMID: 11693522 PMC11337269

[ref111] Sørensen I. Pettolino F. A. Bacic A. Ralph J. Lu F. O’Neill M. A.. (2011). The charophycean green algae provide insights into the early origins of plant cell walls. Plant J. 68, 201–211. doi: 10.1111/j.1365-313X.2011.04686.x, PMID: 21707800

[ref112] Stadnik M. J. Freitas M. B. (2014). Algal polysaccharides as source of plant resistance inducers. Trop. Plant Pathol. 39, 111–118. doi: 10.1590/S1982-56762014000200001

[ref113] Staudacher E. (2012). Methylation—an uncommon modification of glycans. Biol. Chem. 393, 675–685. doi: 10.1515/hsz-2012-0132, PMID: 22944672 PMC3795332

[ref114] Steiner A. A. (1984). “The universal nutrient solution.” in The Sixth International Congress on “Soilless Culture.” Apr 29-May 5; Pudoc, Wageningen, The Netherlands, 633–650.

[ref115] Tan L. Eberhard S. Pattathil S. Warder C. Glushka J. Yuan C.. (2013). An *Arabidopsis* cell wall proteoglycan consists of pectin and arabinoxylan covalently linked to an arabinogalactan protein. Plant Cell 25, 270–287. doi: 10.1105/tpc.112.107334, PMID: 23371948 PMC3584541

[ref116] Temple H. Mortimer J. C. Tryfona T. Yu X. Lopez-Hernandez F. Sorieul M.. (2019). Two members of the DUF579 family are responsible for arabinogalactan methylation in *Arabidopsis*. Plant Direct. 3:e00117. doi: 10.1002/pld3.117, PMID: 31245760 PMC6508755

[ref117] Templeton D. W. Quinn M. Van Wychen S. Hyman D. Laurens L. M. L. (2012). Separation and quantification of microalgal carbohydrates. J. Chromatogr. A 1270, 225–234. doi: 10.1016/j.chroma.2012.10.034, PMID: 23177152

[ref118] Thordal-Christensen H. Zhang Z. Wei Y. Collinge D. B. (1997). Subcellular localization of H_2_O_2_ in plants. H_2_O_2_ accumulation in papillae and hypersensitive response during the barley—powdery mildew interaction. Plant J. 11, 1187–1194. doi: 10.1046/j.1365-313X.1997.11061187.x

[ref119] Torres M. A. Dangl J. L. Jones D. G. (2002). Arabidopsis gp91phox homologues AtrbohD and AtrbohF are required for accumulation of reactive oxygen intermediates in the plant defense response. PNAS 99, 517–522. doi: 10.1073/pnas.012452499, PMID: 11756663 PMC117592

[ref120] Trdá L. Janda M. Macková D. Pospíchalová R. Dobrev P. I. Burketová L.. (2019). Dual mode of the saponin aescin in plant protection, antifungal agent and plant defense elicitor. Front. Plant Sci. 10:1448. doi: 10.3389/fpls.2019.01448, PMID: 31850004 PMC6893899

[ref121] Van de Wouw A. P. Howlett B. J. (2020). Advances in understanding the *Leptosphaeria maculans—Brassica* pathosystem and their impact on disease management. Can. J. Plant Pathol. 42, 149–163. doi: 10.1080/07060661.2019.1643788

[ref122] Van Loon L. C. Rep M. Pieterse C. M. J. (2006). Significance of inducible defense-related proteins in infected plants. Annu. Rev. Phytopathol. 44, 135–162. doi: 10.1146/annurev.phyto.44.070505.143425, PMID: 16602946

[ref123] Vieira H. H. Bagatini I. L. Guinart C. M. Vieira A. A. H. Vieira H. H. Bagatini I. L.. (2016). tufA gene as molecular marker for freshwater Chlorophyceae. Algae 31, 155–165. doi: 10.4490/algae.2016.31.4.14, PMID: 31411734

[ref124] Villa-Rivera M. G. Cano-Camacho H. López-Romero E. Zavala-Páramo M. G. (2021). The role of Arabinogalactan type II degradation in plant-microbe interactions. Front. Plant Sci. 12:730543. doi: 10.3389/fmicb.2021.730543, PMID: 34512607 PMC8424115

[ref125] Waszczak C. Carmody M. Kangasjärvi J. (2018). Reactive oxygen species in plant signaling. Annu. Rev. Plant Biol. 69, 209–236. doi: 10.1146/annurev-arplant-042817-040322, PMID: 29489394

[ref126] Wichard T. Charrier B. Mineur F. Bothwell J. H. Clerck O. D. Coates J. C. (2015). The green seaweed *Ulva*, a model system to study morphogenesis. Front. Plant Sci. 6:72. doi: 10.3389/fpls.2015.00072, PMID: 25745427 PMC4333771

[ref127] Wiesel L. Newton A. C. Elliott I. Booty D. Gilroy E. M. Birch P. R. J.. (2014). Molecular effects of resistance elicitors from biological origin and their potential for crop protection. Front. Plant Sci. 5:655. doi: 10.3389/fpls.2014.00655, PMID: 25484886 PMC4240061

[ref128] Yaich H. Garna H. Besbes S. Paquot M. Blecker C. Attia H. (2013). Effect of extraction conditions on the yield and purity of ulvan extracted from *Ulva lactuca*. Food Hydrocoll. 31, 375–382. doi: 10.1016/j.foodhyd.2012.11.013

[ref129] Yang J. Duan G. Li C. Liu L. Han G. Zhang Y.. (2019). The crosstalks between jasmonic acid and other plant hormone signaling highlight the involvement of jasmonic acid as a core component in plant response to biotic and abiotic stresses. Front. Plant Sci. 10:1349. doi: 10.3389/fpls.2019.01349, PMID: 31681397 PMC6813250

[ref130] Yemm E. W. Willis A. J. (1954). The estimation of carbohydrates in plant extracts by anthrone. Biochem. J. 57, 508–514. doi: 10.1042/bj0570508, PMID: 13181867 PMC1269789

[ref131] Zipfel C. Robatzek S. Navarro L. Oakeley E. Jones J. D. G. Felix G.. (2004). Bacterial disease resistance through flagellin perception in *Arabidopsis*. Nature 428, 764–767. doi: 10.1038/nature02485, PMID: 15085136

