# Supplementary material for: Arabinogalactan Protein-Like Proteins From Ulva lactuca Activate Immune Responses and Plant Resistance in an Oilseed Crop
Source: Front Plant Sci. 2022 May 20;13:893858. doi: 10.3389/fpls.2022.893858 (PMC9164130; doi:10.3389/fpls.2022.893858)
Supplement: Supplementary file 1 [file Data_Sheet_1.docx]

Supplementary Material

# Supplementary Figures and Tables

## Supplementary Figures


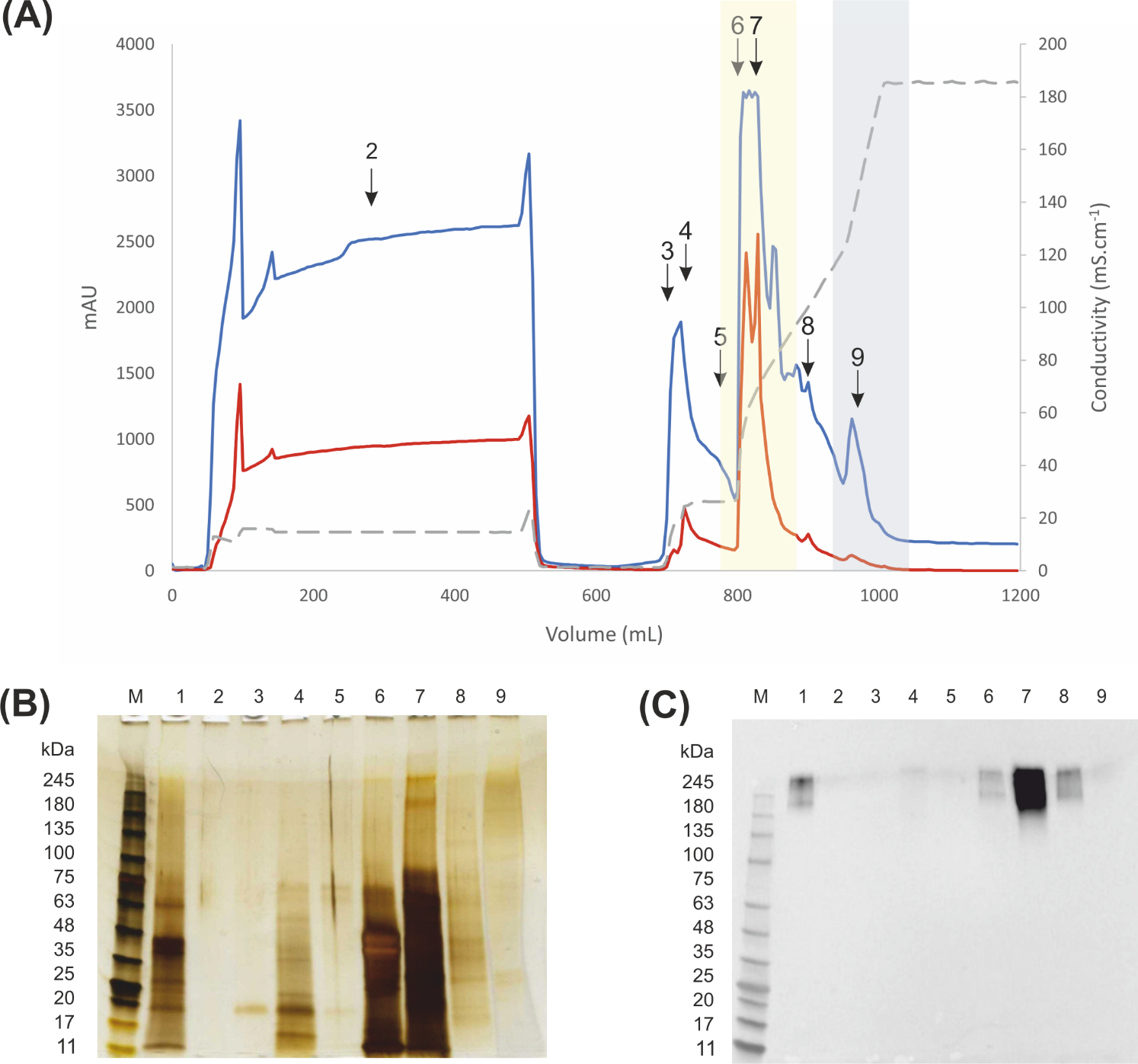


**Supplementary figure 1: (A)** Ion-exchange chromatogram of crude extract (50 mL diluted by 25 mM MES buffer pH 6 ten times) separation on Q FF column. The ionic strength (- - -); absorbance at 210 nm (blue) and absorbance at 280 nm (red). While the yellow area represents the location of JIM16 positive fractions based on western blot, the grey area represents the location of ulvan based on TBO assay. Arrows represent the fractions chosen for subsequent SDS-PAGE and western blot analysis, while the numbers represent the number of the lane on the gel. SDS-PAGE and western blot analysis of ion-exchange chromatography fractions. **(B)** Total protein content visualized by silver staining; **(C)** immunolabeling with anti-AGP JIM16 primary antibody, 30-second exposition time. 1 - Ten times diluted *Ulva lactuca* extract loaded to the column; 2 to 9 - fractions from IEX chromatography. For the SDS-PAGE separation were used 4%-15% gradient precast polyacrylamide gels. Volume loaded on gel: 24 μL of diluted extract and fractions.

**
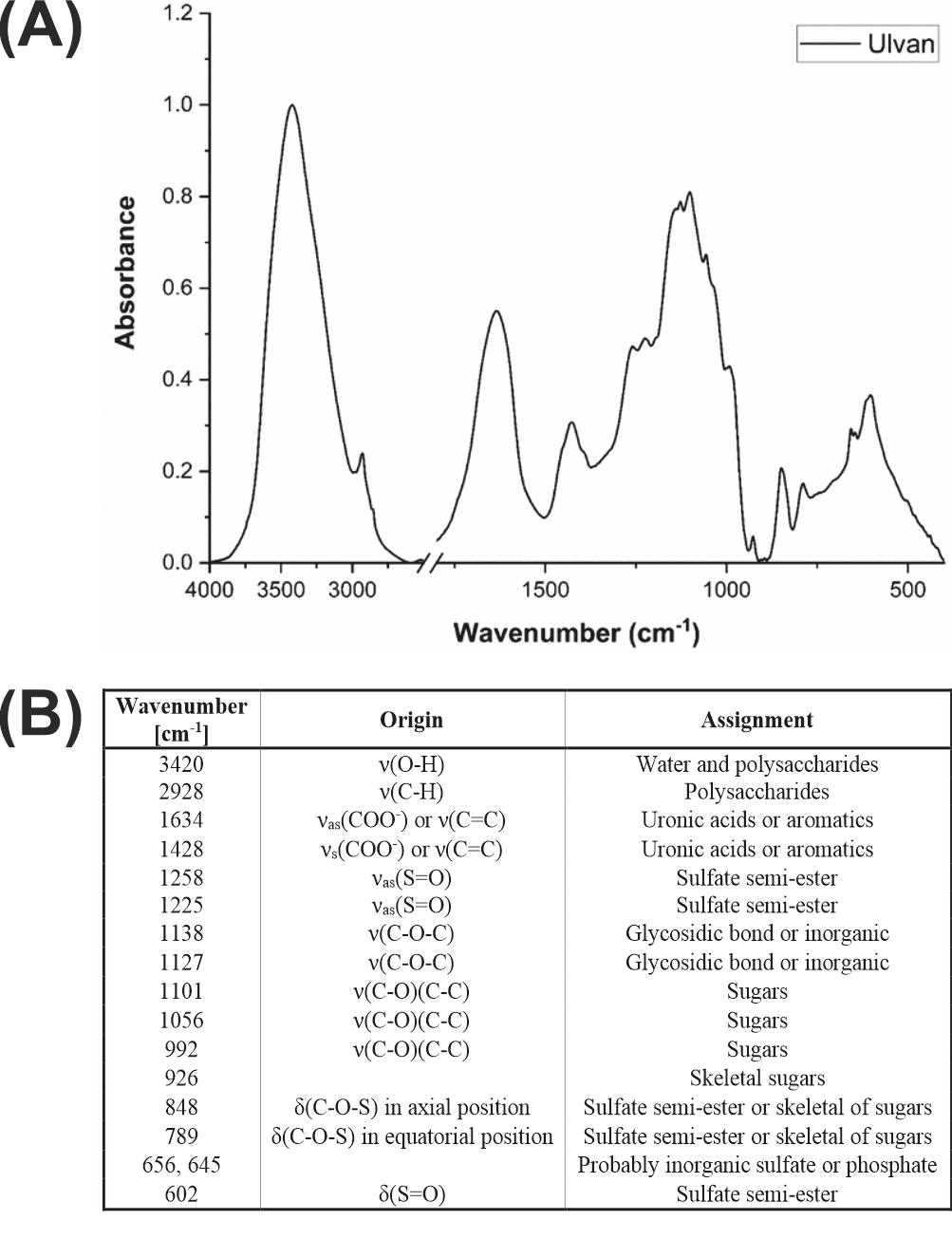
**

**Supplementary figure 2:** FT-IR spectrum of extracted ulvan from *Ulva lactuca* **(A)** and list of present functional groups and their origin **(B)**. Corresponds well to result of Robic et al. (2009).


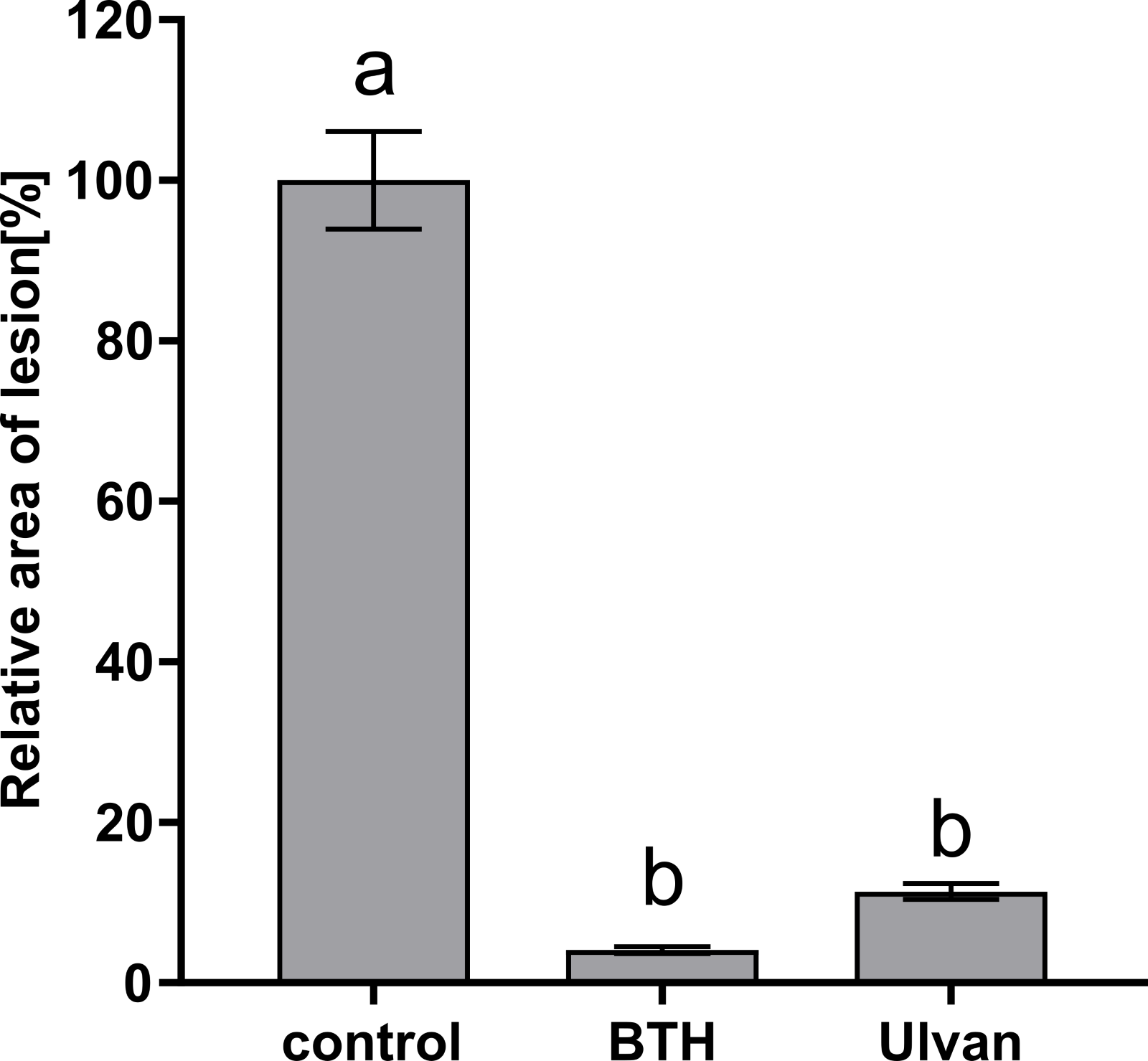


**Supplementary figure 3:** Effect of algal elicitors on the progression of *L. maculans* infection in *B. napus* cotyledons. Cotyledons were treated with ulvan extracted according to Yaich et al. (2013) (Ulvan), distilled water (negative control) and 32 µM BTH (benzothiadiazole, positive control) 2 days before inoculation with *L. maculans*. Disease symptoms were evaluated as a percentage of the lesion area to the leaf area 11 days after inoculation. Ulvan was tested in concentrations 10 mg·mL^-1^. Statistically significant differences determined by the one-way ANOVA and Tukey *post hoc* test (P <0.05). The data are presented as the mean ± SE (n = 24).

## Supplementary Tables

Supplementary table 1: Table of primers

| Gene | EST | Sequence (5'->3') | Amplicon (bp) |
| --- | --- | --- | --- |
| *ACT* | AF111812 | CTGGAATTGCTGACCGTATGAG | 142 bp |
|  |  | TGTTGGAAAGTGCTGAGGGA |  |
| *PR1* | BNU21849 | CATCCCTCGAAAGCTCAAGAC | 90 bp |
|  |  | CCACTGCACGGGACCTAC |  |
| *ICS1* | EV225528 | CAAACTCATCATCTTCCCTC | 192 bp |
|  |  | AGCGTGACTTACTAACCAG |  |
| *PAL1* | DQ341308.1 | GACTAATCTCATCTCGCAAG | 112 bp |
|  |  | ATTCTCCTCCAAGTGTCTTAG |  |
| *ACS2a* | HM450312 | AGGTGGTCAAAGACTTAGATAG | 127 bp |
|  |  | ACCGAGTCGTTGTAAGAATA |  |
| *βCHI* | X61488 | TGCTACATAGAAGAAATAAACG | 118 bp |
|  |  | TTCCATGATAGTTGAATCGG |  |
| *VSP* | CN726858 | CCTCTCACTTTCACTTCTCTTGC | 121 bp |
|  |  | GTTCGGCTTCGTCCTCAATG |  |
| *AOS* | EV124323 | CGCCACCAAAACAACAAAG | 116 bp |
|  |  | GGGAGGAAGGAGAGAGGTTG |  |
| *RD26* | GT085050.1 | ATCGGTCTTTCAATCTTCCT | 193 bp |
|  |  | GAGTTCATCTGCAAATTCCT |  |
| *NCED3* | EV137674 | CGATTTGCCTTACCAAGTCAG | 201 bp |
|  |  | TTTATCCCTTCCGGTGAGAA |  |
| *RBOH D* | EV029129 | TATCCTCAAGGACATCATCAG | 103 bp |
|  |  | TTTCCTCGTCCTAAACCCT |  |
| *RBOH F* | EV035965 | TGTTCTCTTATTGGTTGGTC | 143 bp |
|  |  | TTCCTGTGCTGTTCTCTG |  |
